# The PPEAO experimental fishing dataset: Fish from West African estuaries, lagoons and reservoirs

**DOI:** 10.3897/BDJ.7.e31374

**Published:** 2019-02-14

**Authors:** Monique Simier, Jean-Marc Ecoutin, Luis Tito de Morais

**Affiliations:** 1 MARBEC, Univ Montpellier, CNRS, Ifremer, IRD, Sète, France MARBEC, Univ Montpellier, CNRS, Ifremer, IRD Sète France; 2 LEMAR, IRD, Univ BREST, CNRS, Ifremer, Plouzané, France LEMAR, IRD, Univ BREST, CNRS, Ifremer Plouzané France

**Keywords:** Coastal area, Crustacean, Estuary, Experimental fishing, Fish, Lagoon, Mollusc, Reservoir lake, Tropical area, West Africa

## Abstract

**Background:**

This paper describes a dataset of fish, crustacean and mollusc occurrences extracted from the “Experimental Fishing” section of the IRD's PPEAO information system. PPEAO stands for “Fish communities and artisanal fisheries of West African estuarine, lagoon and freshwater ecosystems”. This database contains information collected using two different methods: experimental fishing and surveys of the artisanal fisheries that exploit these ecosystems. The database is accessible at http://ppeao.ird.fr.

**New information:**

The current dataset is available on GBIF.org at 10.15468/ra4voa. It comprises the occurrences of 314 fish, crustacean and mollusc taxa collected in experimental sampling surveys of different aquatic ecosystems in West Africa between 1979 and 2013. Different types of fishing gear were used including purse seines, gill nets and fyke nets. The taxa were identified by IRD scientists or by scientific partners well trained in systematics. Most taxa were identified at species level (97% of cases). This dataset is the result of 213 fishing surveys, 5,362 fishing hauls and 31,709 occurrences (28,428 of fish taxa and 3,281 of crustaceans and molluscs). The number of individuals per species and per haul is included and 80% of occurrences are geolocated.

## Introduction

### General context

More than 12 million people work in fisheries in Africa, including more than 2.3 million women. Fish represents, on average, 22% of the protein intake in sub-Saharan Africa; in some countries, this level exceeds 50%. For example, the food security of the Senegalese population depends largely on fish resources: nearly 70% of the animal protein consumed is derived from the sea. Senegal is the second largest fish-producing country in West Africa and now exceeds 500,000 tonnes extracted per year. Estuarine and lagoon fish assemblages are of great importance for fisheries in West Africa, both being directly exploited by local fishermen and constituting nursery areas for many marine species targeted by coastal fisheries.

Knowledge and monitoring of biodiversity is therefore an important issue in West Africa, both in terms of knowledge of aquatic environments and the assessment of aquatic resources for sustainable use. The ecosystem approach to fisheries advocated by FAO particularly requires this type of information. The dataset presented here is of special importance in this context because it combines both old data (the taxonomy of which have been reviewed in the light of current knowledge) and recent data, thus allowing an approach to the temporal evolution of biodiversity. This database is the largest experimental fisheries catch database for West Africa and covers both a large temporal range (more than three decades) and a large spatial range in West Africa (12 ecosystems in seven countries). It comprises fish occurrences mainly from estuarine and lagoon ecosystems (Ebrié lagoon, Sine Saloum estuary including Bamboung MPA, Fatala estuary, Rio Grande de Buba, Gambia estuary), but also from coastal ecosystems (Bijagos archipelago including Urok Island MPA, Banc d'Arguin National Park and Dangara inlet) and also two reservoir lakes (Manantali and Selingue).

After having developed the PPEAO information system, we then felt it was important to provide it with greater visibility and accessibility for the international scientific community. GBIF.org is probably the major global portal for the provision of this type of data, which justifies the choice made here to use it.

### The PPEAO information system

Starting in the early 1960s, the French «*Office de la Recherche Scientifique et Technique Outre-Mer*» (ORSTOM), which in 1998 became the «*Institut de Recherche pour le Développement*» (IRD), studied West African aquatic ecosystems. These studies included sampling of aquatic communities by scientists and surveys of artisanal fisheries that exploit the ecosystems. Several research programmes focused on various estuarine, lagoon, coastal and lake ecosystems in West Africa involving the same scientific team succeeded one another between 1979 and 2013. From 2001 onwards, the IRD research programme “Adaptative responses of fish populations and assemblages to the environmental pressures” (“*Réponses Adaptatives des populations et des peuplements de Poissons aux pressions de l’environnement*”, French acronym RAP) funded the development of two MS-Access databases and of the software needed for their management and for user consultation: “Pechart” for the artisanal fisheries database (Ndiaye et al. 2003a) and “Pechexp” for the experimental fishing database (Ndiaye et al. 2003b). In these two databases, historical data (1980-2000) was gathered in a homogeneous format along with new data collected in the RAP research programme between 2001 and 2008. Then, in 2006, to make the data available to a wider scientific community, the two databases were merged into one PostgreSQL database, which is located on an IRD server. The development of this database, called PPEAO for “*Peuplements de poissons et Pêche artisanale des Ecosystèmes estuariens, lagunaires et continentaux d'Afrique de l'Ouest*” and of its management and consultation software, were funded by IRD, through the support project “*Soutien aux Projets Informatiques dans les équipes Scientifiques*” (SPIRALES – Ecoutin et al. 2007). PPEAO software can be accessed at http://ppeao.ird.fr.

### The dataset available on GBIF.org

A first dataset gathering all the species occurrences observed by experimental fishing was extracted from PPEAO and put online on GBIF.org. It is available at 10.15468/ra4voa. The present datapaper describes this dataset, which corresponds to 213 fishing surveys, 5,362 fishing hauls and 31,709 occurrences (28,428 of fish taxa and 3,281 of crustaceans and molluscs). The number of individuals per species and per haul is available and 80% of occurrences are geolocated. In the following section, we provide a general description of the dataset, after which we give a more detailed description of each ecosystem with their respective sampling protocols.

## General description

### Purpose

**Figure 1.**
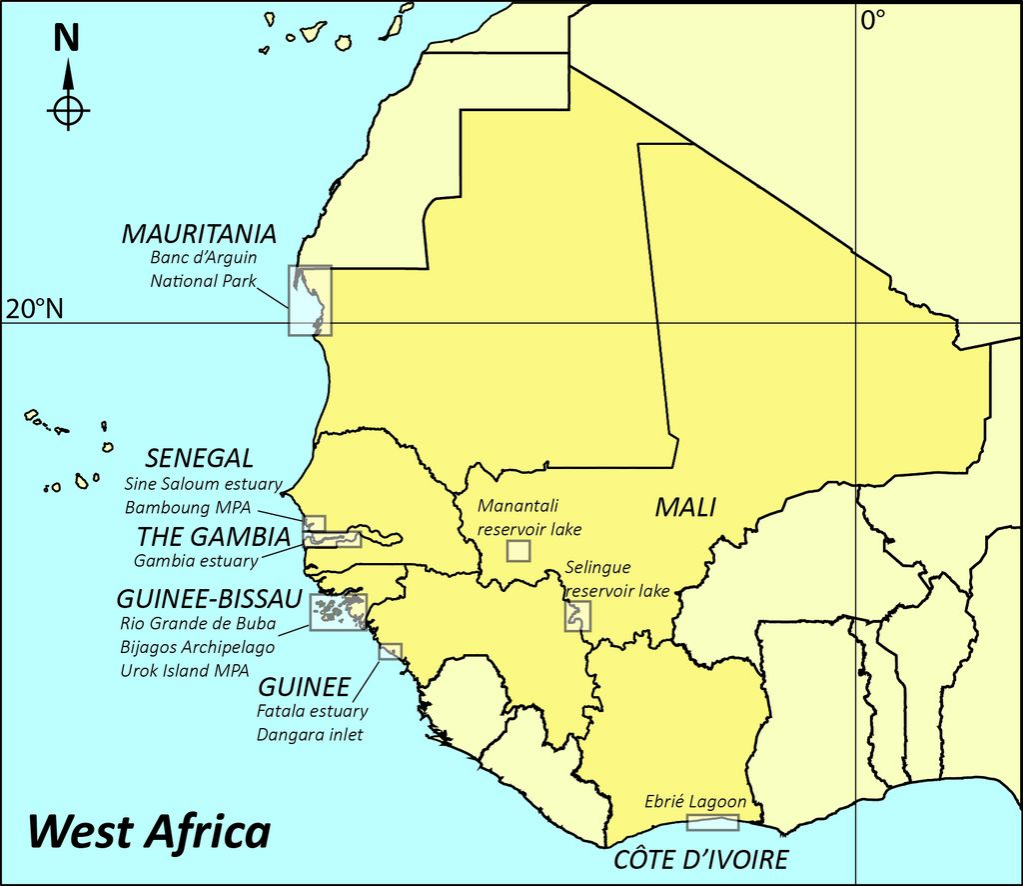
Map of West Africa showing the location of the study areas (boxes).

This dataset covers several aquatic ecosystems in West Africa (estuaries, lagoons and reservoirs – Fig. 1): “historical” data collected since 1979, data sampled by the IRD RAP research unit between 2000 and 2008 and recent data, i.e. up to 2013 (Table 1). The taxa are mainly fish and a few crustaceans and molluscs.

## Project description

### Title

The project PPEAO “Fish communities and artisanal fisheries of West African estuarine, lagoon and freshwater ecosystems” was started in 2006 by the former IRD research unit “Adaptative responses of fish populations to the environmental pressures” (“*Réponses Adaptatives des populations de poissons aux pressions de l'environnement*”, French acronym RAP).

### Personnel

**Main team (in alphabetical order)**: Jean-Jacques Albaret (sample collection, sample identification), Jean-Marc Ecoutin (sample collection, data management), Raymond Laë (project coordinator), Jean Raffray (sample collection, sample identification), Oumar Sadio (sample collection, sample identification), Monique Simier (data management), Luis Tito de Morais (sample collection, sample identification), Guy Vidy (sample collection, sample identification).

**Other collectors**: Eric Baran (Guinea), Famara Darboe (Gambia), Itaf Deme-Gningue (Senegal), Papa Samba Diouf (Senegal)

### Study area description

**General spatial coverage**: West Africa (Fig. 1)

**Coordinates**: 4°N and 21°N Latitude; 18°W and 2°W Longitude

### Design description

The sampling surveys completed and embedded in the PPEAO database had different scientific objectives, for example, describing the fish community of an aquatic ecosystem and its space-time variability, searching for particular species, comparing and calibrating biological indicators, testing new sampling devices, demonstrating fishing techniques or carrying out evaluations. In the first case, a sampling protocol was defined, with particular fishing gear and sites that were regularly sampled during each survey.

### Funding

IRD, Institut de Recherche pour le Développement, France

## Sampling methods

### Study extent

This dataset covers several aquatic ecosystems in West Africa (estuaries, lagoons and reservoirs – Fig. 1): “historical” data collected since 1979, data sampled by the IRD RAP research unit between 2000 and 2008 and recent data, i.e. up to 2013 (Table 1). The taxa are mainly fish and a few crustaceans and molluscs.

### Sampling description

**Methods**: The sampling unit was the fish haul defined by: a site (station), a date (survey), the fishing gear used. After each fishing haul, all the individuals captured (fish, crustaceans, molluscs) were identified, if possible at the species level. All the individuals captured in the same haul and belonging to the same species were counted.

**Sampling**: Each geographical entity studied was considered as an ecosystem. In each ecosystem, a number of sampling sites was defined. Data collection consisted of several fishing surveys using one or several fishing gears. The most frequently used fishing gear (58% of occurrences) was a purse seine. It was 250 m long, 18 m deep with a 14 mm mesh size. The purse seine was used blindly, without searching for fish in the defined sampling sites. Other types of fishing gear were used: fyke nets (22.6% of occurrences) to sample juvenile fish, gill nets (12%) in Guinea (in addition to purse seine), Mali and the Urok islands MPA, trawl (5.7%) in the deepest area of the Ebrié lagoon and beach seines (1.6%) in the Sine Saloum and the Bijagos.

### Quality control

Each sampling survey was supervised by a scientist trained in systematics. Amongst them were IRD senior scientists who contributed to reference papers or books about West African fish species (*e.g. Albaret 2003, Albaret 2017, Albaret and Desfossez 1988, Polidoro et al. 2017, Polidoro et al. 2016*). Oumar Sadio, the junior scientist who identified fish species for all the recent surveys, was trained in fish identification during three months in the Royal Museum for Central Africa in Tervuren (Belgium). This enabled precise identification of the collected taxa. Most taxa were identified at species level (97% of cases).

## Geographic coverage

### Description

West Africa (Fig. 1)

### Coordinates

4°N and 21°N Latitude; 18°W and 2°W Longitude.

## Taxonomic coverage

### Description

The main aim of samplings for this dataset was to describe the fish communities. Consequently, fish account for the majority of observed taxa (278 out of 314), but some crustaceans, molluscs and cnidarians were also observed (18, 17 and 1 individual, respectively). Amongst the 278 fish taxa, 261 were identified at the species level, representing 98.6% of the occurrences, 14 at the genus level (0.8% of the occurrences) and 3 at the family level (0.6% of the occurrences). Fish taxonomy is given according to the Catalog of Fishes Online Database (Fricke et al. 2018), the authoritative reference for taxonomic fish names. Table 2 lists the fish taxa ordered by family with the total number of individuals collected per country.

Despite the high variability of the sampling effort in the different countries (from 3 surveys in Mauritania to 93 surveys in Senegal), the total number of families identified per country was stable, ranging from 40 to 60, except in Mali where only 14 families were observed (Table 3). The number of taxa was also low in Mali (50 taxa), whereas it ranged from 71 in Guinea-Bissau (5 surveys) to 141 in Senegal (93 surveys).

**Figure 2.**
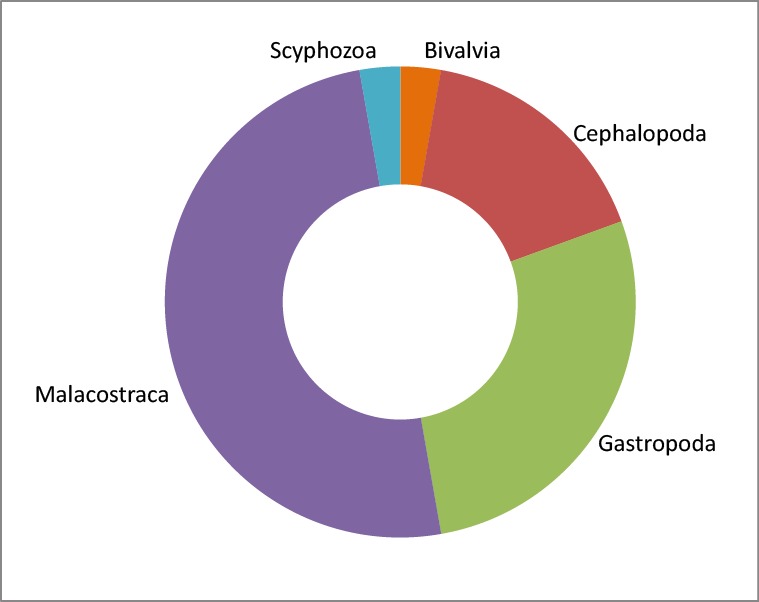
Proportion per class (in number of taxa) of taxa other than fish in the PPEAO database. Bivalvia : 1 taxa, Cephalopoda : 6 taxa, Gastropoda : 10 taxa, Malacostraca : 18 taxa, Scyphozoa : 1 taxa.

As mentioned above, fish assemblages were the main target of experimental fishing surveys. However, other groups (crustaceans, molluscs and cnidaria) were caught accidentally and, in some ecosystems, recorded in the database. Their taxonomy is given according to the World Register of Marine Species (WoRMS Editorial Board 2019). These 36 taxa are listed in Table 4. They account for a total of 3,821 occurrences, identified at different levels: 74% at species level (24 species), 16.7% at genus level (8 genera), 2.5% at family level (1 family), 4.8% at order level (2 orders) and 1.8% at class level (1 class). Crustaceans in the Malacostraca class accounted for half of the total number of observed taxa other than fish (Fig. 2), while molluscs accounted for almost all the other half (10 Gastropoda, 6 Cephalopoda and 1 Bivalvia), except for one Scyphozoa, only identified at the class level.

## Temporal coverage

**Data range:** 1979-12-17 – 2013-5-15.

## Usage rights

### Use license

Other

### IP rights notes

This work is licensed under a Creative Commons Attribution Non Commercial (CC-BY-NC) 4.0 License

## Data resources

### Data package title

The PPEAO experimental fishing dataset: Fish from West African estuaries, lagoons and reservoirs

### Resource link

https://www.gbif.org/dataset/41f549a6-c7ae-4460-8b2b-3333b1a036cd

### Number of data sets

1

### Data set 1.

#### Data set name

PPEAO - Pêches scientifiques. Peuplements de poissons des écosystèmes estuariens, lagunaires et continentaux d'Afrique de l'Ouest

#### Data format

Darwin Core Archive format

#### Number of columns

57

#### Character set

UTF-8

#### Download URL

http://ipt.gbif.fr/archive.do?r=ppeao-pechexp

#### Data format version

1.0

#### 

**Data set 1.** 

Column label	Column description
id	The unique identifier of the Occurrence
modified	The most recent date-time on which the resource was changed
collectionID	An identifier for the collection or dataset from which the record was derived
institutionCode	The name (or acronym) in use by the institution having custody of the object(s) or information referred to in the record
collectionCode	The name, acronym, coden or initialism identifying the collection or dataset from which the record was derived
basisOfRecord	The specific nature of the data record
informationWithheld	Additional information that exists, but that has not been shared in the given record
dataGeneralizations	Actions taken to make the shared data less specific or complete than in its original form
occurrenceID	The unique identifier of the Occurrence (=id)
catalogNumber	A unique identifier for the record within the dataset or collection
recordNumber	An identifier given to the Occurrence at the time it was recorded
recordedBy	A list (comma separated) of names of people responsible for recording the original Occurrence. The primary collector or observer is listed first
individualCount	The number of individuals represented present at the time of the Occurrence
organismQuantity	A number or enumeration value for the quantity of organisms
organismQuantityType	The type of quantification system used for the quantity of organisms
sex	The sex of the biological individual(s) represented in the Occurrence
establishmentMeans	The process by which the biological individual(s) represented in the Occurrence became established at the location
associatedMedia	A list (concatenated and separated) of identifiers (publication, global unique identifier, URI) of media associated with the Occurrence
samplingProtocol	The name of the method or protocol used during an Event
eventDate	The date when the Event was recorded (dd/mm/yyyy)
eventTime	The time when the Event was recorded (hh:mn:ss)
year	The four-digit year in which the Event occurred, according to the Common Era Calendar
month	The ordinal month in which the Event occurred
day	The integer day of the month on which the Event occurred
habitat	A category or description of the habitat in which the Event occurred
eventRemarks	Comments or notes about the Event
continent	The name of the continent in which the Location occurs
waterBody	The name of the water body in which the Location occurs
country	The name of the country or major administrative unit in which the Location occurs
countryCode	The standard code for the country in which the Location occurs
stateProvince	The name of the next smaller administrative region than country (state, province, canton, department, region etc.) in which the Location occurs
locality	The specific description of the place
minimumElevationInMeters	The lower limit of the range of elevation (altitude, usually above sea level), in metres
maximumElevationInMeters	The upper limit of the range of elevation (altitude, usually above sea level), in metres
minimumDepthInMeters	The lesser depth of a range of depth below the local surface, in metres
maximumDepthInMeters	The greater depth of a range of depth below the local surface, in metres
decimalLatitude	The geographic latitude (in decimal degrees, using the spatial reference system given in geodeticDatum) of the geographic centre of a Location. Positive values are north of the Equator, negative values are south of it. Legal values lie between -90 and 90, inclusive
decimalLongitude	The geographic longitude (in decimal degrees, using the spatial reference system given in geodeticDatum) of the geographic centre of a Location. Positive values are east of the Greenwich Meridian, negative values are west of it. Legal values lie between -180 and 180, inclusive
geodeticDatum	The ellipsoid, geodetic datum or spatial reference system (SRS) upon which the geographic coordinates given in decimalLatitude and decimalLongitude are based
coordinateUncertaintyInMeters	The horizontal distance (in metres) from the given decimalLatitude and decimalLongitude describing the smallest circle containing the whole of the Location
coordinatePrecision	A decimal representation of the precision of the coordinates given in the decimalLatitude and decimalLongitude
identifiedBy	A list (comma separated) of names of people who assigned the Taxon to the subject
dateIdentified	The date on which the subject was identified as representing the Taxon
typeStatus	A list (concatenated and separated) of nomenclatural types (type status, typified scientific name, publication) applied to the subject
scientificName	The full scientific name, with authorship and date information if known
kingdom	The full scientific name of the kingdom in which the taxon is classified
phylum	The full scientific name of the phylum or division in which the taxon is classified
class	The full scientific name of the class in which the taxon is classified
order	The full scientific name of the order in which the taxon is classified
family	The full scientific name of the family in which the taxon is classified
genus	The full scientific name of the genus in which the taxon is classified
subgenus	The full scientific name of the subgenus in which the taxon is classified
specificEpithet	The name of the first or species epithet of the scientificName
infraspecificEpithet	The name of the lowest or terminal infraspecific epithet of the scientificName, excluding any rank designation
taxonRank	The taxonomic rank of the most specific name in the scientificName
scientificNameAuthorship	The authorship information for the scientificName formatted according to the conventions of the applicable nomenclaturalCode
vernacularName	A common or vernacular name

## Additional information

### Detailed description of the dataset

We now provide a detailed description of the climate, geography, sampling protocol and main characteristics of the assemblages in each monitored ecosystem in chronological order. In the case of the Sine Saloum delta, which was the target for several research projects from the 1990s to the 2000s, all the sampling periods are included in the same section. Conversely, the Bamboung MPA, situated in the Sine Saloum delta, was the subject of a particular monitoring programme and is described separately. Sampling in 1993 in Guinea-Bissau and monitoring of the Urok Islands MPA between 2011 and 2013 are also described separately because they had different objectives.

### Ebrié lagoon (Côte d'Ivoire)

Sample collectors: Jean-Jacques Albaret, Jean-Marc Ecoutin and Jean Raffray.

**Figure 3.**
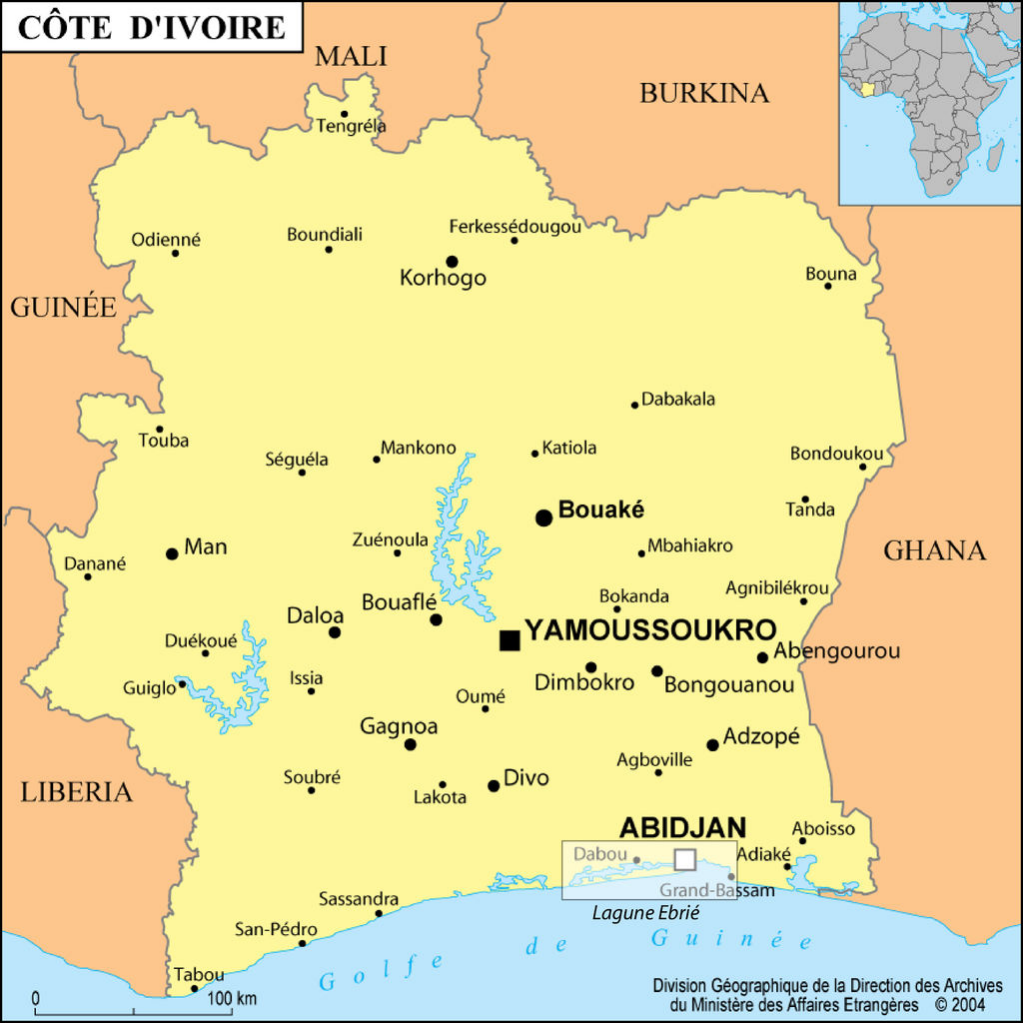
Map of Côte d’Ivoire and location of Ebrié lagoon (box).

The largest coastal lagoon in West Africa extends around Abidjan, over approximately 130 km along the coast of Côte d’Ivoire (Fig. 3). It is located between 5.2° and 5.45° North and 3.7° and 4.8° West. It never exceeds 7 km in width for an average depth of 4.8 m and a surface area of 566 km² (Durand et al. 1994). This lagoon is subject to a double influence: freshwater influence from rainfall and river flow, especially from the Comoé River situated at the East end of the lagoon and marine influence via the Vridi canal which provides permanent access to the sea. There are three distinct seasons: a dry season from January to April, a rainy season from May to August and a flood season from September to December (Durand and Chantraine 1982).

In the 1970s, a comprehensive research programme on the environment and the aquatic resources of the Ebrié lagoon was conducted (Durand et al. 1994). This included a study of the fish assemblages of the lagoon conducted by scientists from Orstom and “*Centre de Recherches Océanographiques*” of Abidjan, from December 1979 to August 1982. The aim of the study was to describe the distribution and functioning of fish assemblages in relation to changes in environmental conditions in space and over time. The results are summarised in a book on environment and aquatic resources of Côte d’Ivoire (Durand et al. 1994) by Albaret (1994) and in a more recent article by Ecoutin et al. (2005). Several other publications, based on these data, describe the ecology and the biology of the main lagoon fish species.

Based on preliminary studies, the Ebrié lagoon was divided into six sectors. The Vridi canal separates two dissymetric parts: sectors I and II are located in the east part and sectors IV to VI in the west part. In the centre, sector III is directly influenced by the Atlantic Ocean via the Vridi canal. In the sampling protocol of the fish assemblages, around 80 sampling sites were defined covering all six sectors.

Two kinds of sampling gear were used: trawls and purse seines. The trawls had the following characteristics: vertical opening 1.5 m – headrope 10 m – mesh size 20 mm. The 137 trawl hauls were only conducted in sector III, the most maritime area of the Ebrié lagoon, from December 1979 to August 1982. The purse seine (300 m long, 18 m deep with a 14 mm mesh size) was used to sample the whole Ebrié lagoon in a total of 406 hauls. The most extreme sectors (I, V and VI) were only sampled in two seasons: in the middle of the dry season (February and March 1981) and at the end of the rainy season – beginning of the flood season (October 1980 and August-September 1981). The central sectors (II, II and IV) were sampled at approximately two-monthly intervals, alternating between the west and the east part of the lagoon, between February 1980 and October 1981. Finally, the bay of Cocody, located in the town of Abidjan, was sampled monthly with a purse seine from October 1980 to October 1981 and the results were published by Albaret and Ecoutin (1990).

Sampling of Ebrié lagoon recorded 6,537 fish occurrences, corresponding to 60 families and 113 taxa, all identified at the species level (Table 5). The Clupeidae family clearly dominated the fish assemblages, with *Ethmalosa fimbriata* accounting for more than 70% of the occurrences, followed by *Sardinella maderensis* (7%). Other families were also well represented: Carangidae with *Chloroscombrus chrysurus* (2.8%), Claroteidae with *Chrysichthys maurus* and *Chrysichthys nigrodigitatus* (2.4 and 1.6%, respectively), Paralichthyidae with *Citharichthys stampflii* (2.1%) and Gerreidae with *Gerres nigri* and *Eucinostomus melanopterus* (2.1 and 0.9%, respectively).

During these sampling campaigns, 16 taxa other than fish were recorded, corresponding to 1,007 occurrences: 69% of the individuals were identified at the species level, 24% at the genus level, 0.5% at the family level and 6% at the class level. They were mainly penaeid shrimps *Penaeus notialis*, jellyfish (*Scyphozoa*) and crabs belonging to the genus *Callinectes*.

### Sine Saloum estuary (Senegal)

Sample Collectors: Jean-Jacques Albaret, Papa Samba Diouf, Jean Raffray, Oumar Sadio, Luis Tito de Morais and Guy Vidy.

**Figure 4.**
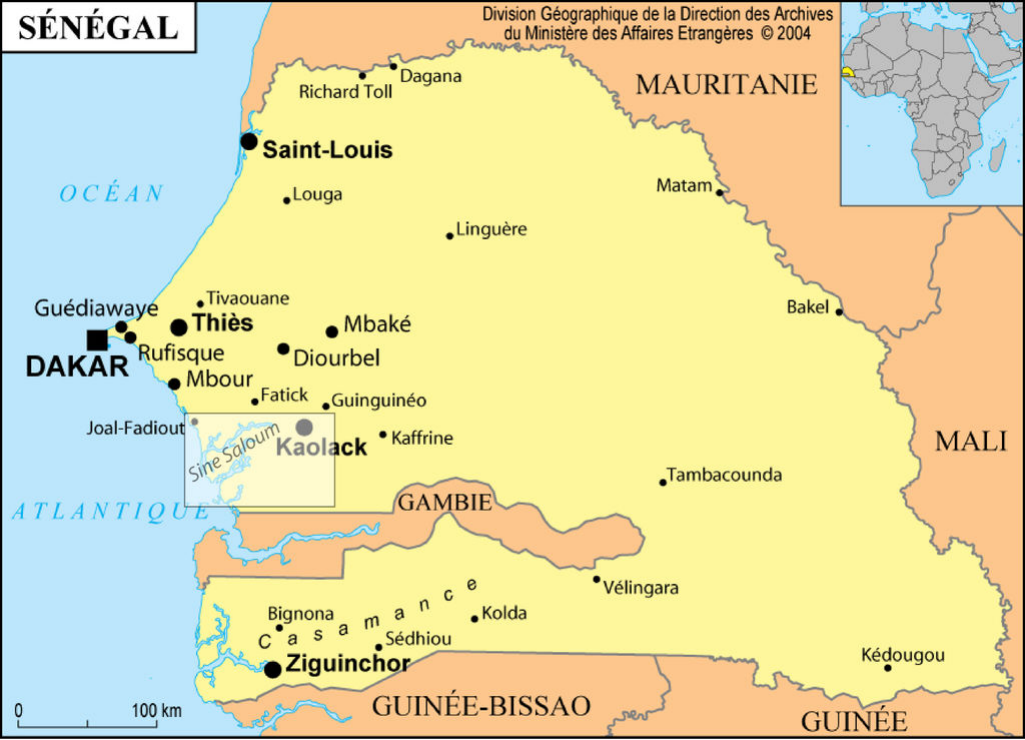
Map of Senegal showing the location of the Sine-Saloum delta (box).

The Sine Saloum delta is located 100 km south of Dakar and north of the Gambia River and Casamance estuaries between 13.6° and 14.2° North and 15.8° and 16.8° West (Fig. 4). It has three main branches from north to south: the Saloum, the Diomboss and the Bandiala. These branches are shallow (10 to 15 m deep in the centre of the channel, with a maximum depth of 25 m in some places). The branches are interconnected by a dense network of seawater creeks locally named “bolongs”, resulting in two groups of islands, partially covered with mangrove. Eight ecological zones have been defined in the Sine Saloum delta (Diouf 1996): Z1 to Z3 in the Bandiala, Z4 in the Diomboss, considered as homogeneous and Z5 to Z8 in the Saloum. The estuary drains a watershed of 29,720 km². The Sudanese type climate is characterised by two seasons: a short, wet and warm season from July to October and an extended dry season, cool from November to March and warm from April to June. Due to the very low gradient of the watershed and the lack of rain, the Sine Saloum is an inverse estuary, i.e. salinity increases upstream, particularly in the Saloum branch. The fish assemblages in this delta have been studied in several research programmes since the early 1990s.

**The 1990s**

In the early 1990s, a research programme was conducted in the Sine Saloum delta by Orstom and the “*Centre de Recherche Océanographique de Dakar Thiaroye*” (CRODT), part of the “*Institut Sénégalais de Recherches Agricoles*” (ISRA), with the collaboration of the “*Ecosystèmes Lagunaires*” laboratory belonging to Montpellier University, France. This research programme was funded by Orstom. The dataset contains data collected between 1990 and 1997 by the two components of the programme. The “adult and subadult fish assemblages” component was conducted by Jean-Jacques Albaret and the results were published by Diouf (1996) and later by Simier et al. (2004). For the “juvenile fish assemblages” component, conducted by Guy Vidy (Vidy 2000), the dataset specifically comprises data collected using fyke nets.

Two types of fishing gear were used to sample adult and subadult fish assemblages: beach seines and purse seines. Samplings using a “long” beach seine (180 m long, 9 m deep with a 25 mm mesh) were conducted in April, June and September 1990. In December 1990, a few trials were conducted using a “truncated” beach seine and a 100 m long purse seine. From April 1991 to May 1993, a standardised sampling protocol was used with a purse seine 250 m long, 18 m deep with a 14 mm mesh size. Ten sampling sites were defined covering the eight zones of the Sine Saloum delta. On each sampling occasion, each site was sampled twice, one haul in the middle of the channel and the other near the bank, to obtain a representative picture of the diverse habitats. All the sites were sampled at two or three monthly intervals, except the two sites in zone Z8, upstream of the Saloum, which were sampled only twice: once during the wet season (October 1992) and once during the dry season (February 1993). A few “off-protocol” surveys were also done, using the same purse seine, but without following the standardised protocol. Surveys carried out in July, August and September 1994 only covered small “bolongs” fish assemblages. Finally, a last survey was carried out in January 1997 following the standardised protocol.

The study of juvenile fish assemblages began at the end of 1994 and ended at the beginning of 1997 (Vidy 2000). Only the mangrove area was sampled. After a period of adjustment of the sampling technique and gear, the survey lasted two years. The main sampling gear was a fyke net 2.5 m long and 0.4 m in diameter, inspired by the “*capéchades*” used in the South of France. The net was completed by two wings each 1.5 m long and by a 4 m long “wall” to guide the fish towards the trap. The mesh size was 6 mm for the wall and 3 mm for the second half of the trap. Sampling took place every month at night during the new moon phase, at flood tide to catch the fish entering the mangrove corridors. Two successive strategies were used. In 1995, three sites were sampled on two consecutive nights, with four traps, giving an eight samples set per site and per month. This period was dedicated to the characterisation of seasonal variability of the juvenile fish community. In 1996, three more sites were added and the six sites were sampled on only one night using six traps. The aim of this strategy was to obtain details on the spatial organisation of juvenile fish.

**Early 2000s**

In the early 2000s, the Sine Saloum delta was chosen by the RAP research unit for a comparative study with the nearby Gambia River estuary. The research programme was funded by IRD. The aim of this comparative study of the fish assemblages between the inverse Sine Saloum estuary and the normal Gambia River estuary was to better understand the impact of a major climatic disturbance, hypersalinity. For this reason, more samplings were conducted in the hypersaline upstream area of the Saloum than in the 1990s.

Concerning the sampling of adult and subadult fish assemblages in the main channels, a preliminary survey was conducted in March 2002, followed by three surveys using the same standardised protocol: in May 2002 (dry and warm season), October 2002 (wet and warm season) and March 2003 (dry and cool season). During each survey, 30 purse seine hauls were done at 18 sampling sites (13 in the Saloum, one in the Diomboss and four in the Bandiala). As in the 1990s, most of the sites were sampled twice on a given date: one haul in the mid-channel and the other one close to the bank. In the Saloum, six sites were sampled only once per survey, as well as the two Bolong Dioto sites in the Bandiala. The sampling gear was a purse seine 250 m long, 18 m deep with a 14 mm mesh size. The results were published by Ecoutin et al. (2010). Two additional surveys, not using the standardised protocol, were carried out in 2004.

The main fishing gear used for the sampling of juvenile fish assemblages was a fyke net, which was also used in the Gambia River estuary during the same period (Vidy et al. 2004). The gear comprised two parts: the first was a “wall” 8 m long, 3 m high with an 8 mm mesh size. The “wall” was used to guide the fish towards the second part of the trap, the fyke net itself, 3 m long and 0.4 m in diameter, with two wings each 2.5 m long. The mesh size of the fyke net was 6 mm. For each survey, eight traps were deployed at each site for one night, in or near the mangrove, at a maximum depth of 3 m. Fish sampling was carried out in the new moon phase, at flood tide, in order to catch the fish entering the mangrove corridors.

**Years 2005-2006**

In 2005 and 2006, scientists of the RAP research unit chose to focus on the biological indicators of the state of health of fish populations and assemblages. The aim was to develop a set of indicators that could be analysed jointly to assess the extent of a disturbance in aquatic ecosystems. This work was conducted in the context of the GIBAO project (*Groupement Indicateurs Biologiques d’Afrique de l’Ouest*). It was funded by IRD, with financial help from the Sub-Regional Fisheries Commission (CSRP) of West Africa, as part of the Regional Partnership for Coastal and Marine conservation (PRCM).

Targeted operations were conducted in the form of purse seine fish samplings. Six sampling sites were defined, one in the uspstream area of the Bandiala (zone 3), the five others in the Saloum (zones 6, 7 and 8), not including the mouth area. During each survey, between 3 and 9 stations were sampled per site. Samplings were made using a purse seine 250 m long, 18 m deep with a 14 mm mesh size. All the hauls were classified as “off protocol” because they did not cover the entire Sine Saloum delta.

**General description of catch data**

The consecutive research programmes in the Sine Saloum delta recorded 8,567 fish occurrences, in all the periods combined, corresponding to 56 families and 126 taxa, 95.5% of them being identified at the species level, 2.5% at the genus level and 2% at the family level (Table 6). The Clupeidae family dominated the assemblages, 35% of the occurrences were *Sardinella maderensis* and 22% *Ethmalosa fimbriata*. Cichlidae were well represented with three species: *Sarotherodon melanotheron* (10% of occurrences), *Hemichromis fasciatus* and *Coptodon guineensis* (less abundant with 0.4 and 0.3% occurrences, respectively). Pristigasteridae *Ilisha africana* accounted for 8.7% of occurrences, while Haemulidae *Brachydeuterus auritus* and Carangidae *Chloroscombrus chrysurus* accounted for 3.2 and 2.3% of occurrences, respectively. Gerreidae, with *Gerres nigri* (5%) and *Eucinostomus melanopterus* (3.3%) and Mugilidae (*Chelon dumerili*, *Neochelon falcipinnis*, *Parachelon grandisquamis*, *Mugil cephalus* and *Mugil curema*) also represented a significant component (3.6%) of the fish assemblages in the Sine Saloum delta.

During the samplings in the Sine Saloum delta, 969 occurrences representing 14 taxa other than fish were also recorded, of which 66.7% were identified at the species level, 18.3% at the genus level, 7.9% at the family level and 7% at the order level. Most of the occurrences corresponded to shrimps *Penaeus notialis* and crabs *Callinectes amnicola*. Some cephalopoda (Loliginidae and *Sepia*) were also quite often collected.

### Fatala Estuary & Dangara inlet (Guinea)

Sample Collectors: Jean-Jacques Albaret, Eric Baran.

**Figure 5.**
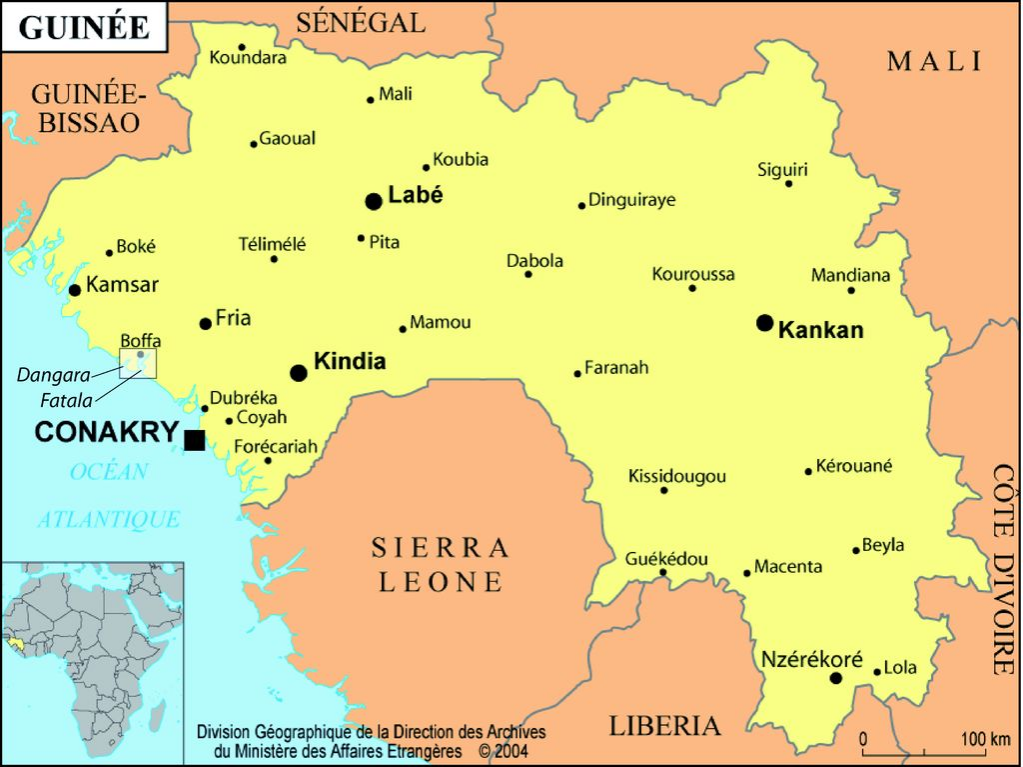
Map of Guinea showing the location of the Fatala estuary and the Dangara inlet (box).

In order to assess the influence of the riverine system on the estuarine ichthyfauna, a comparative study was conducted in Guinea between January 1993 and March 1994. The aim of the study was to compare the fish assemblages in the estuary of the Fatala River with those in the neighbouring Dangara inlet, located 20 km away and not connected to a river. This study was conducted by Eric Baran for his PhD thesis (Baran 1995), which was directed by Christian Lévêque and advised by Jean-Jacques Albaret. The whole research programme was funded by Orstom, in cooperation with the inland fisheries department of the “*Centre National des Sciences Halieutiques de Boussoura*” (CNSHB). The study area was located in the coastal zone of Boffa, which belongs to the “Southern Rivers” region (Fig. 5). It is located between 10° and 10.3° North and 14° and 14.25° West. The tropical wet climate is characterised by a dry season from November to May and a wet season from June to October with peak rainfall in July. Rainfall is very high (approximately 3,500 mm/year) with the hydrological cycle reaching a maximum in August or September (Baran 1995).

In the Fatala estuary, the estuarine zone is limited by a rock sill 60 km upstream which stops both the dynamic and saline actions of the tide. As the area 10 km upstream of the estuarine zone could not be accessed by boat, sampling was limited to the downstream 50 kilometres. Two fishing gears, a purse seine and gillnets, were used alternately every month, to obtain a comprehensive description of fish assemblage variability. The purse seine (250 m long, 18 m deep with a 14 mm mesh size) was used every two months from January 1993 to January 1994, at eight equidistant sites, one of which was located in the ocean in front of the river mouth. Each site was sampled during daylight, each time with two hauls, one haul in the mid-channel and the other as near as possible to the bank (minimum water level: 2 m). The gillnets were set up in parallel and close to the bank, in order to provide a better sampling of riparian fish assemblages. A gillnet set was composed of ten panels of different mesh size (10, 12.5, 15, 17.5, 20, 22.5, 25, 30, 35 and 40 mm). Each panel was 25 m long and 2 m deep. Four sampling sites, the same as those used for the purse seine samplings, were located 3, 17, 33 and 46 km from the mouth, respectively. At each site, four “stations” were defined, corresponding to two subsequent nights on both sides of the estuary. In the Dangara inlet, three sites were defined. Dangara surveys took place immediately before or after those in the Fatala estuary, i.e. every two months. Only purse seine samplings were performed, twice at each site as in the Fatala estuary (mid-channel and close to the bank).

Together, the samplings performed during this study in Guinea recorded 3,910 fish occurrences, corresponding to 40 families and 86 taxa, 99.8% of which being identified at the species level and 0.2% at the genus level (Table 7). The Pristigasteridae *Ilisha africana* dominated the fish assemblages with 35.3% of the occurrences, followed by the Sciaenidae *Pseudotolithus elongatus* (14%) and the Carangidae *Chloroscombrus chrysurus* (8.5%). Clupeidae *Sardinella maderensis*, *Ethmalosa fimbriata* and *Pellonula leonensis* represented 8.7%, 7.1% and 4.4% of the occurrences, respectively. Haemulidae *Brachydeuterus auritus* accounted for 4.2% of the occurrences. Mugilidae were rare in the Fatala estuary and the Dangara inlet, except for *Neochelon falcipinnis* and *Parachelon grandisquamis* (2% each). The same applies to the Gerreidae family, which was only represented by *Eucinostomus melanopterus* (0.7%).

Only fish were collected during this study and no information is available about taxa other than fish.

### Bijagos Archipelago & Rio Grande de Buba (Guinea-Bissau)

Sample Collectors: Jean-Jacques Albaret, Papa Samba Diouf and Itaf Deme-Gningue.

**Figure 6.**
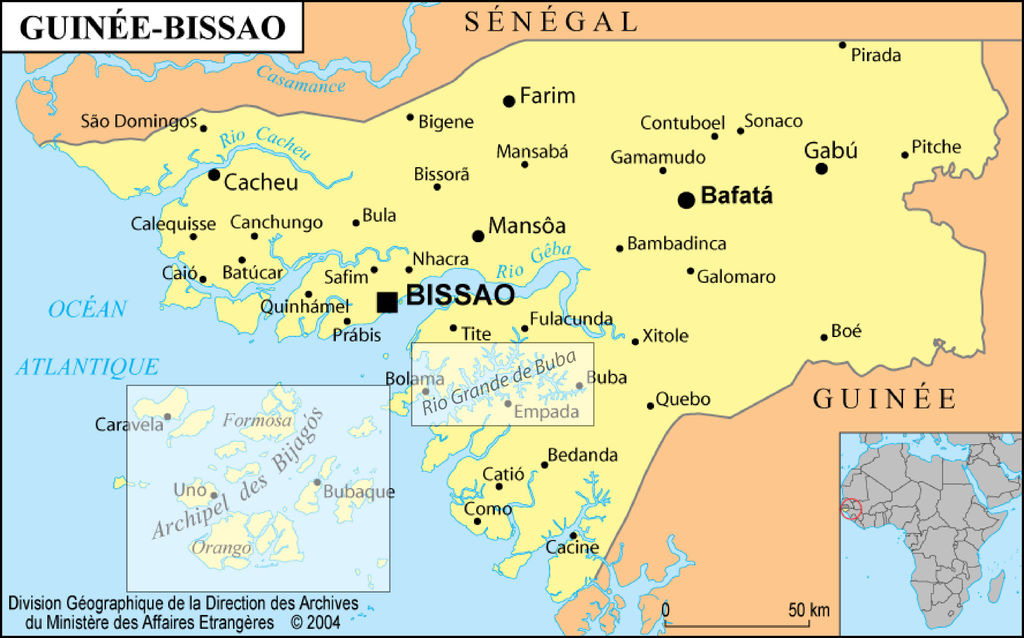
Map of Guinea-Bissau showing the location of the two areas sampled in 1993: the Bijagos archipelago and the Rio Grande de Buba (boxes).

The Bijagos Archipelago is located off the coast of Guinea-Bissau between 10.9° and 11.6° North and 15.6° and 16.5° West. The Rio Grande de Buba is a small coastal river flowing in the west central region of Guinea-Bissau, between 11.4° and 11.7° North and 15° and 15.5° West. It flows into the Atlantic Ocean in front of the Bijagos Archipelago (Fig. 6).

Following a request from the “*Centre Canadien d’Etudes et de Coopération Internationale*” (CECI), a team from Orstom and the “*Centre de Recherche Océanographique de Dakar Thiaroye*” (CRODT - Sénégal) carried out a scientific survey in the Bijagos Archipelago at the end of March 1993. This team was composed of Itaf Deme-Gningue and Pape Samba Diouf from CRODT and Jean-Jacques Albaret from Orstom. Scientists and technicians from the Fisheries Department of Guinea-Bissau and from CECI also took part in the survey. Following this survey, a second survey was carried out by the same team in the Rio Grande de Buba at the begining of April 1993 in response to a request from the International Union for Conservation of Nature (IUCN). Jean-Louis Kromer (UICN) participated in this survey. The results of the surveys were published in two scientific reports (Deme-Gningue et al. 1994 and Diouf et al. 1994).

In the Bijagos Archipelago and the Rio Grande de Buba, a sampling plan of 36 and 24 sites, respectively, was followed, considering the diversity of habitats and bathymetry. The GPS positions were recorded for the Bijagos Archipelago, but not for the Rio Grande de Buba. The main fishing gear used was a purse seine (250 m long, 18 m deep with a 14 mm mesh size). At some sites, a beach seine was used to provide information about riparian fish communities. It was used either instead of the purse seine (at sites 25 and 30 in the Bijagos Archipelago) or in addition to it (at sites 32 and 34 in the Bijagos Archipelago and sites 1 and 10 in the Rio Grande de Buba). This beach seine was 50 m long, 2 m depth with a 30 mm size for the wings and 14 mm mesh for the bunt.

The two surveys performed in 1993 in the Bijagos Archipelago and the Rio Grande de Buba in Guinea-Bissau recorded 258 fish occurrences, corresponding to 43 families and 60 taxa; all fish were identified at the species level (Table 8). The Carangidae *Chloroscombrus chrysurus* dominated the assemblages with 51.5% of occurrences. The Gerreidae *Eucinostomus melanopterus* and *Gerres nigri* accounted for 17.6 and 5.4% of occurrences, respectively. The two Clupeidae *Sardinella maderensis* and *Ethmalosa fimbriata* accounted for 7.7% and 1.4% and the Pristigasteridae *Ilisha africana* 2.1%, respectively.

During these two surveys, 28 occurrences of taxa other than fish were identified. They belonged to five taxa, mainly shrimp *Penaeus notialis* and crabs belonging to the genus *Callinectes*.

### Gambia estuary (Gambia)

Sample Collectors: Jean-Jacques Albaret, Famara Darboe, Jean, Luis Tito de Morais and Guy Vidy.

At the begining of the 2000s, the Gambia River estuary was chosen by the RAP research unit of IRD as a reference ecosystem for a comparison of West African estuarine fish assemblages. The Gambia River estuary has rarely been disturbed by drought and functions like a “normal” estuary, with no upstream dams; it is surrounded by an abundant mangrove in good condition, with little pollution and subject to low fishing pressure.

**Figure 7.**
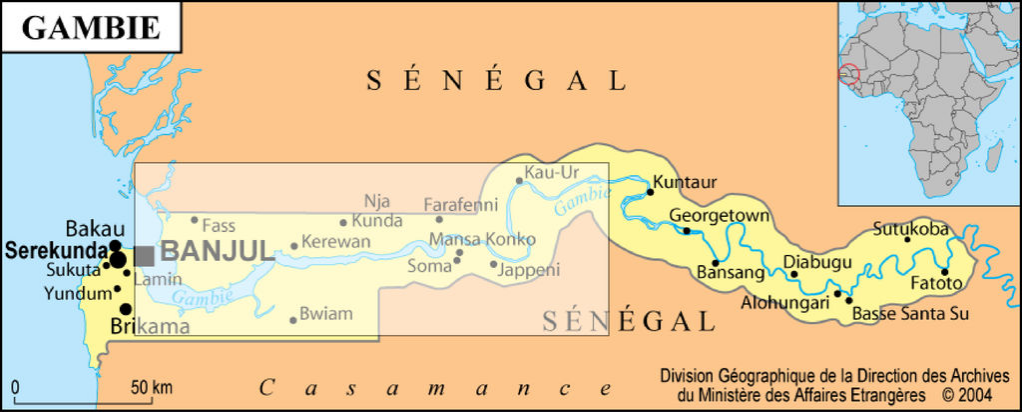
Map of Gambia showing the location of the estuarine zone of the river sampled in 2000-2002 (box).

A joint IRD/Gambian Fisheries Department research programme was conducted from 2001 to 2003, for the “Evaluation of the fish resources and estuarine resources stewardship of the Gambia River”. It was funded by the French Department for Cultural Action and Co-operation (SCAC). The study area comprised the lower, middle and upper estuary, from Banjul at the mouth to Deer Island 220 km upstream (Fig. 7). The Gambia estuary is located between 13.2° and 13.7° North and 15° and 16.6° West. In this region, the wet season extends from June to October, with peak rainfall in August. The cool dry season lasts from November to March and the warm dry season from April to June.

Five surveys were conducted to characterise the adult and subadult fish assemblages in the main stream of the Gambia River estuary, covering the whole hydrological cycle. Not all the subsequently sampled sites were visited during the preliminary survey (November 2000), whereas six of the originally envisaged sites were abandoned after the preliminary survey. During each of the four following surveys (June 2001, September 2001, December 2001 and April 2002), 44 sites were sampled, only using a purse seine (250 m long, 18 m deep with a 14 mm mesh size). The results were published by Albaret et al. (2004) and Simier et al. (2006).

Eight surveys were conducted between September 2001 and November 2003 to study mangrove juvenile fish assemblages. The first two surveys in 2001 enabled the sampling protocol to be adjusted. Then, in 2002 and 2003, three surveys per year were conducted between May and November, the season of maximum juvenile recruitment, at six sites along the estuary. The main gear used to sample juvenile fish was the same as that used in the Sine Saloum delta during the same period (see above). The results were published by Vidy et al. (2004).

Together, the samplings performed in the Gambia River estuary recorded 3,710 fish occurrences, corresponding to 40 families and 92 taxa, almost all identified at the species level (Table 9). The Sciaenidae *Pseudotolihus elongatus* was predominant, accounting for 35.8% of the occurrences, followed by the Clupeidae *Ethmalosa fimbriata* (22.1%) and the Pristigasteridae *Ilisha africana* (11.7%). Another Clupeidae, *Sardinella maderensis*, accounted for 6.4% of the occurrences and the Mugilidae Parachelon *grandisquamis* for 3.5%. Unlike the other ecosystems studied, three freshwater fish were identified amongst the 20 main fish taxa in the Gambia River estuary: the two Mochokidae *Synodontis gambiensis* (3.4%) and *Synodontis batensoda* (1.1%) and the Schilbeidae *Schilbe intermedius* (0.6%).

During the survey of the Gambia River estuary, 893 occurrences of taxa other than fish were recorded. They corresponded to 10 taxa, mostly *Callinectes amnicola* and *Penaeus notialis*.

### Selingué and Manantali reservoirs (Mali)

Sample Collectors: Jean-Marc Ecoutin, Raymond Laë, Jean Raffray and Luis Tito de Morais.

**Figure 8.**
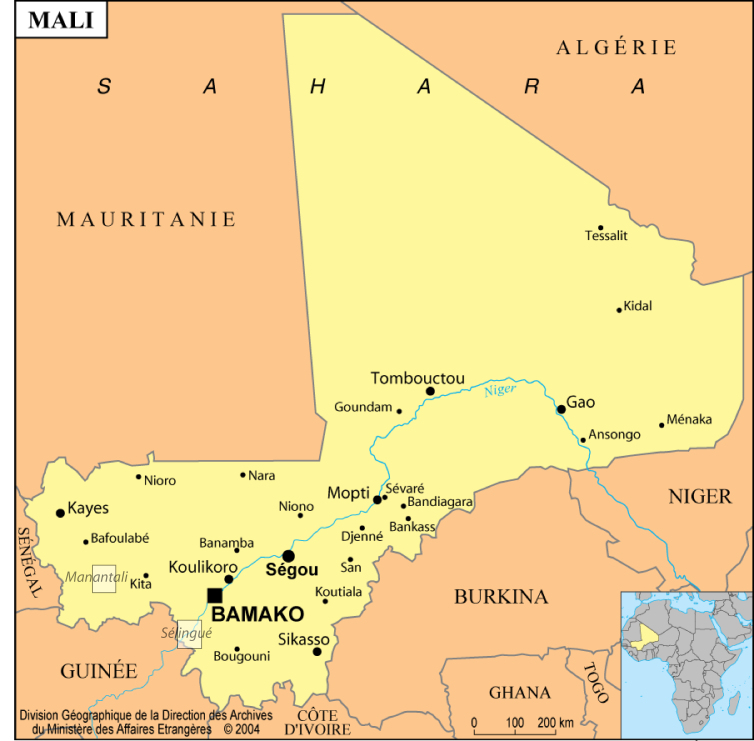
Map of Mali showing the location of the two reservoirs surveyed in 2002-2003: Manantali and Sélingué (boxes).

In 2002 and 2003, a comparative study was conducted by the RAP research unit of IRD in Mali, to better understand the impact of fishing effort on fish assemblages. This study was funded by the Priority Solidarity Fund (*Fonds de Solidarité Prioritaire* - FSP), part of the French Ministry of Foreign Affairs. The Manantali and Sélingué man-made reservoirs (Fig. 8) were selected because of their similar size and edaphic and environmental properties, but subject to different degrees of fishing pressure: low at Manantali vs. high at Sélingué (Kantoussan 2007). Manantali is located between 12.9° and 13.3° North and 10.2° and 10.4° West and Sélingué is located between 11.2° and 11.6° North and 8° and 8.4° West. Three methods of assessment were combined for this study: fish gillnet sampling, surveys of artisanal fisheries and hydroacoustics (Coll et al. 2007).

Regarding fish gillnet sampling, three surveys were conducted in both reservoirs: a preliminary survey in June 2002, which enabled adjustment of the sampling protocol and two surveys covering the key hydrological seasons: the end of the dry season (June 2003) and the end of the rainy season (October 2003), corresponding to the minimum and the maximum water levels, respectively. A detailed description of the sampling protocol is given in Laë et al. (2006) and Coll et al. (2007). Three zones were defined *a priori* in Manantali reservoir: the dam, the middle zone and the upper zone. The same zones were defined in Sélingué reservoir, separating Bale and Sankarani, the two rivers flowing into the lake. Due to limited accessibility, time and high costs, only two areas in each reservoir were actually sampled: the dam and central zones in Manantali and the dam and central zones on the Sankarani side in Sélingué (Coll et al. 2007).

Sampling was carried out using monofilament gillnet panels with five different mesh sizes (10, 15, 22.5, 45 and 80 mm) joined together to form a gang. Each single mesh panel was 25 m long and 3 m deep. Each gang therefore comprised a fishing area of 375 m² (Coll et al. 2007).

Together, the samplings performed in the Manantali and Sélingué reservoirs recorded 1,266 fish occurrences, corresponding to 14 families and 50 taxa, almost all identified at the species level (Table 10). The number of families was particularly low compared to the other ecosystems studied, mostly estuaries and lagoons. It is indeed a freshwater environment with depauperate fauna because of the arid sahel environment and even for Africa freshwater, it is a species poor area. The fish assemblages were also very different, with a strong freshwater component, typically Nilo-Sudanic. Alestidae accounted for a large proportion (42.8%) of the occurrences, with predominantly *Hydrocynus forskahlii* (24.5%), *Brycinus leuciscus* (11.2%) and *Brycinus nurse* (6.2%). The Schilbeidae family came in second place (15.9%), with mainly *Schilbe mystus* (13.6%). Cyprinidae came in third place (12.5%) with *Enteromius macrops* and *Labeo coubie*, representing 7.2 and 2.3% of the occurrences, respectively. The Claroteidae *Chrysichthys auratus* represented 6.6% and the Cichlidae *Hemichromis fasciatus* and *Hemichromis bimaculatus* 3.2 and 2.5%, respectively.

During these samplings, only fish were collected and no taxa other than fish were recorded.

### Bamboung MPA (Senegal)

Sample collectors: Jean-Jacques Albaret, Jean Raffray, Oumar Sadio and Luis Tito de Morais.

This dataset is the result of the biological survey of the Bamboung Marine Protected Area (MPA) in Senegal located 130 km south of Dakar, between 13.75° and 13.84° North and 16.5° and 16.56° West, in the Biosphere Reserve of the Sine Saloum Delta (Fig. 4). The Bamboung bolong is a small affluent of the Diomboss, one of the main branches of the Sine Saloum Delta. The MPA covers 68 km², with a wetland zone composed of mangrove and savannah and a water body of approximately 4 km². It is one of the sites chosen for the purpose of demonstration in the “*Narou Heuleuk*” (tomorrow’s share) project. It was proposed and driven by the Oceanium, a Senegalese organization for the protection of marine resources, and originally funded by the French Fund for the World Environment (*Fond Français pour l’Environnement Mondial* - FFEM). The biological monitoring of the Bamboung MPA, was implemented by the RAP research unit of IRD and funded from 2008 to 2011 by the French National Research Agency (http://www.agence-nationale-recherche.fr/?Projet=ANR-07-BDIV-0009) as part of the AMPHORE project “Marine Protected Areas: biodiversity conservation tools and sustainable management of fishery resources” (“*Les Aires Marines Protégées: outil de conservation de la biodiversité et de gestion durable des ressources halieutiques”).* From 2009 to 2012, this study was part of the CEPIA project of the Sub-Regional Fisheries Commission (*Commission Sous-Régionale des Pêches* - CSRP) entitled “Building together a fisheries management integrating MPA” (“*Construire Ensemble une gestion des Pêches Intégrant les AMP*”), funded by the French Development Agency (AFD) and coordinated by the International Union for Conservation of Nature (IUCN). The Bamboung bolong became a MPA in December 2003, was monitored from March 2003 to December 2012, leading to the publication of a summary report (Ecoutin et al. 2013), and several articles, two of which specifically concerned fish assemblages (Ecoutin et al. 2014; Sadio et al. 2015).

A purse seine (250 m long, 18 m deep with a 14 mm mesh size) was used for all fish samplings. Sampling was conducted three times a year: in March (dry and cool season), in May/June (dry and warm season) and in September/October (wet season). Two different protocols were followed successively. From 2003 to 2007, only the MPA was sampled at 12 sites distributed throughout the 15 km of the bolong. The three surveys conducted in 2003 enabled a reference status to be defined for fish assemblages and the following surveys made it possible to characterize their evolution after the creation of the MPA. From 2008 to 2012, only six sites were kept in the MPA, while for the purpose of comparison, six other sites were selected in the neighboring Sangako bolong, located upstream and subject to fish exploitation, along with four sites in the Diomboss channel, between Bamboung and Sangako bolongs, a total of 16 sites. Finally, in the last year of the study (2012), four additional sites were sampled in the Diomboss channel, downstream from the Bamboung MPA.

Together, the samplings performed in the Bamboung bolong and its surroundings (including Sangako bolong and Diomboss channel) recorded 3,247 fish occurrences, corresponding to 48 families and 97 taxa, almost all identified at the species level (Table 11). The Clupeidae *Ethmalosa fimbriata* (42%) and *Sardinella maderensis* (34.6%) clearly dominated the assemblages, followed by the Carangidae *Chloroscombrus chrysurus* (4.6%), the Mugilidae *Chelon dumerili* (2.9%) and the Pristigasteridae *Ilisha africana* (2.7%). The Gerreidae *Gerres nigri* and *Eucinostomus melanopterus* were also well represented (2.6 and 2.5%, respectively).

During this study, 333 occurrences belonging to 16 taxa other than fish were collected; 76.5% were identified at the species level and 23.1% at the genus level. Amongst them, a large number of molluscs were identified (*Hexaplex duplex*, *Senilia senilis* etc.), as well as crustaceans (*Callinectes amnicola* and *Penaeus notialis)*.

### The Banc d’Arguin National Park (Mauritania)

Sample collectors: Raymond Laë and Luis Tito de Morais.

**Figure 9.**
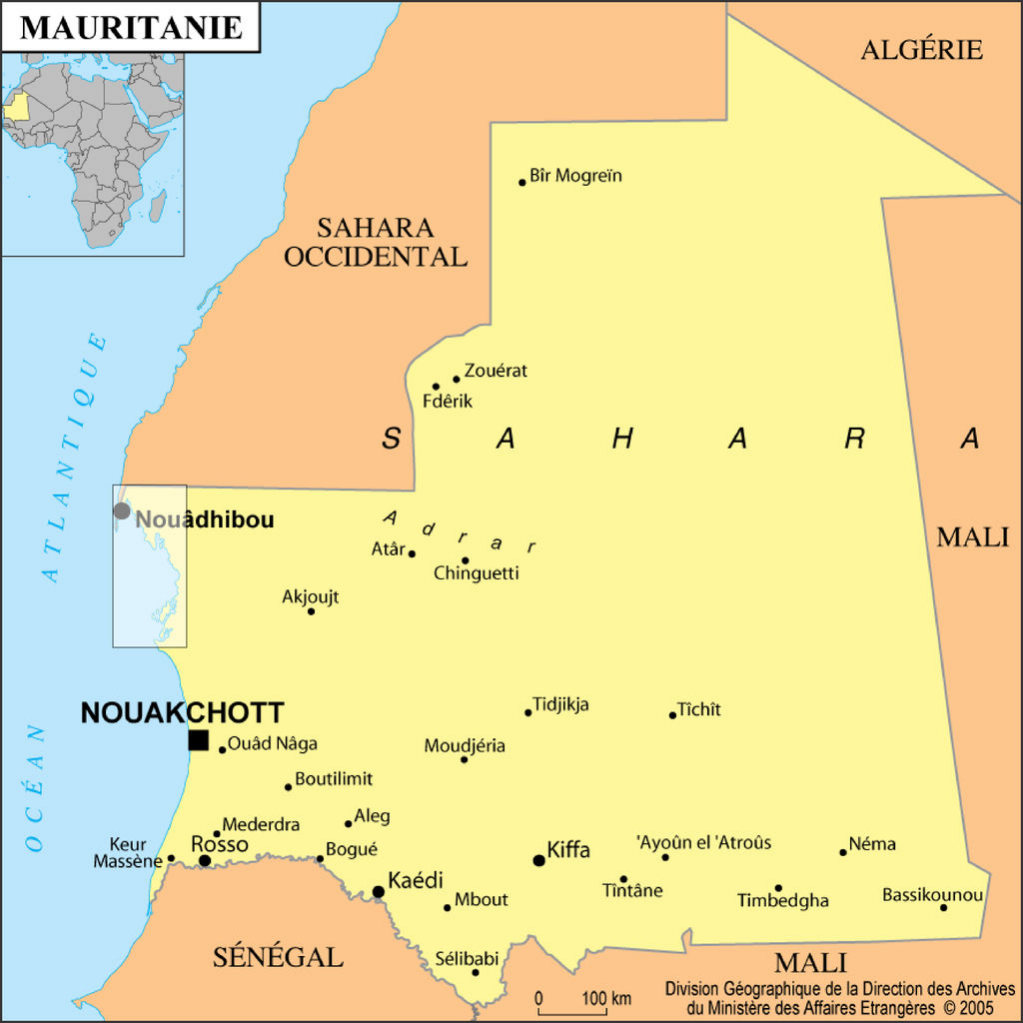
Map of Mauritania showing the location of the Banc d’Arguin National Park (box).

Although the term “MPA” did not yet exist, the first West African MPA was the Banc d’Arguin National Park in Mauritania. The Banc d’Arguin is a coastal ecosystem located on the edge of the Sahara desert, between 19.4° and 20.8° North and 16° and 16.8° West (Fig. 9). Created in 1976, the Banc d’Arguin National Park was classified as a “Ramsar Site” (Ramsar Convention on Wetlands of International Importance, especially as Waterfowl Habitat) in 1982, then added to the UNESCO World Heritage List in 1989. It is the largest MPA in West Africa, with more than 12,000 km² equally divided between a marine and a terrestrial zone (Sadio 2015).

The Banc d’Arguin National Park was sampled as part of the AMPHORE project “Marine Protected Areas: biodiversity conservation tools and sustainable management of fishery resources” (“*Les Aires Marines Protégées: outil de conservation de la biodiversité et de gestion durable des ressources halieutiques*”) funded from 2008 to 2011 by the French National Research Agency (http://www.agence-nationale-recherche.fr/?Projet=ANR-07-BDIV-0009). A purse seine (250 m long, 18 m deep with a 14 mm mesh size) was used to sample fish assemblages by a team of IRD scientists, in cooperation with the Mauritanian Institute of Oceanographic Research and Fisheries (IMROP). Thirty four sites were defined inside and outside the MPA. Some had to be cancelled because they were not accessible by boat. Two surveys were conducted in 2008, in the dry season (7-14 May) and the wet season (8-13 October). A third survey began on 27 April 2010, but had to stop after the first four hauls due to technical problems, before starting again from 22 -28 May 2010. The wet season survey could not be carried out in 2010 (Sadio 2015).

The three surveys performed in the Banc d’Arguin National Park recorded 530 fish occurrences, corresponding to 41 families and 84 taxa, almost all identified at the species level (Table 12). The fish assemblages were dominated by three families: Clupeidae (43.5%), Sparidae (28.2%) and Carangidae (19.5%). Clupeidae were mainly represented by *Sardinella maderensis* (26.7%), *Sardinella aurita* (12.2%) and *Ethmalosa fimbriata* (4.6%), Sparidae by *Diplodus bellottii* (26.3%) and Carangidae by *Chloroscombrus chrysurus* (18.8%). The Monacanthidae *Stephanolepis hispidus* was also frequently recorded (2.5% of the occurrences).

During these samplings, 44 occurrences of 12 taxa other than fish were recorded. They were either identified at the species level (66%) or the genus level (34%). The most frequent taxa belonged to the genera *Murex* and *Sepia* and to the species *Penaeus notialis*.

### Urok Islands MPA (Guinea-Bissau)

Sample collectors: Jean-Marc Ecoutin and Oumar Sadio.

**Figure 10.**
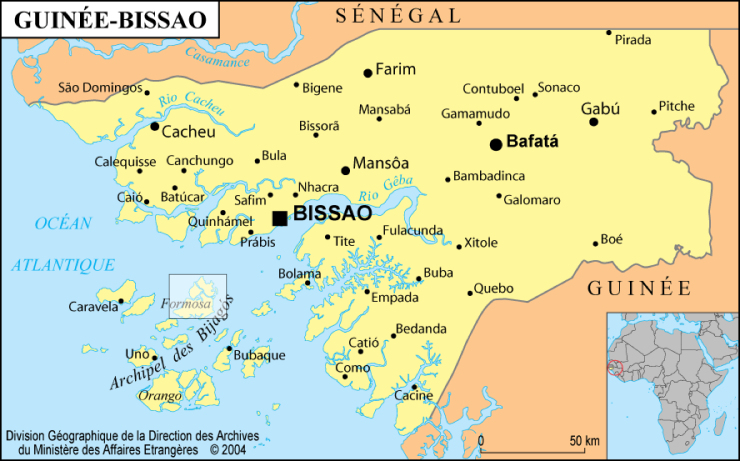
Map of Guinea-Bissau showing the location of the Urok Island MPA (box).

The Urok Islands complex is located in the coastal area of Guinea-Bissau, in the northern part of the Bijagos Archipelago biosphere reserve (Fig. 10). The Marine Protected Area of the Urok Islands was created in 2005, after agreement was reached between the resident populations, the NGO Tiniguena and the administrative authorities of Guinea-Bissau.

The Urok Islands MPA covers 545 km², 398 km² of which is in the maritime zone. It is located between 11.4° and 11.6° North and 15.9° and 16.1° West. The MPA is broken down into three zones, where fishing is authorised at different levels. The aim of zoning was to allow the central zone, where the fishing restrictions are the strictest, to have both attraction and spillover effects. This study was part of the CEPIA project of the Sub-Regional Fisheries Commission (*Commission Sous-Régionale des Pêches* - CSRP) entitled “Building together a fisheries management integrating MPA” (“*Construire Ensemble une gestion des Pêches Integrant les AMP”)*, funded by the French Development Agency (AFD) and coordinated by the International Union for Conservation of Nature (IUCN). The sampling protocol was designed and implemented by a team of IRD scientists (Sadio 2015). The surveys were conducted in cooperation with the *Instituto Nacional de Pesquisa Agraria* (INPA) and the NGO Tiniguena, using both bottom and surface gillnets. Each set of gillnets was made up of six panels with different mesh sizes (14, 25, 36, 50, 60 and 80 mm). Each panel was 25 m long and 2 m deep. The gillnets were set up at high tide, with a fishing depth of at least 7 m. The surveys were carried out in November 2011 and 2012 at the end of the rainy season and in May 2013, at the end of the dry season (Sadio 2015).

The three surveys performed in the Urok Islands MPA recorded 403 fish occurrences, corresponding to 30 families and 47 taxa, all identified at the species level (Table 13). Fish assemblages were dominated by the Ariidae family, with *Carlarius latiscutatus* accounting for 29.9% and *Carlarius parkii* for 9.9%. The Pristigasteridae *Ilisha africana* and the Haemulidae *Pomadasys perotaei* each represented 5.4% of the occurrences. The Carcharhinidae *Rhizoprionodon acutus* was also often observed (4.6% of the occurrences).

During these samplings, only seven occurrences of taxa other than fish were recorded.

## Figures and Tables

**Table 1.** Ecosystems represented in the PPEAO experimental fishing dataset (in chronological order), including the country, the number of surveys, the number of hauls, the sampling period and the geographical coordinates.

**Ecosystem**	**Country**	**Surveys Number**	**Number of hauls**	**Start of sampling**	**End of sampling**	**Latitude range (North)**	**Longitude range (West)**
Ebrié lagoon	Côte d’Ivoire	73	543	Dec 17,1979	Aug 31, 1982	5.2° – 5.45°	3.7° – 4.8°
Sine Saloum estuary	Sénégal	63	1416	Apr 20,1990	Oct 26, 2007	13.6° – 15.8°	15.8° – 16.8°
Fatala estuary	Guinea	13	1164	Jan 22, 1993	Mar 22, 1994	10° – 10.3°	14° – 14.25°
Dangara inlet	Guinea	7	65	Jan 29 1993	Jan 24, 1994		
Bijagos Archipelago	Guinea-Bissau	1	43	Mar 23, 1993	Apr 1, 1993	10.9° – 11°6	15.6° – 16.5°
Rio Grande de Buba	Guinea-Bissau	1	26	Apr 3, 1993	Apr 7, 1993	11.4° – 11.7°	15° – 15.5°
Gambia estuary	Gambia	13	562	Nov 24, 2000	Nov 24, 2003	13.2° – 13.7°	15° – 16.6°
Selingue reservoir lake	Mali	3	371	Jun 10, 2002	Oct 15, 2003	11.2° – 11.6°	8° – 8.4°
Manantali reservoir lake	Mali	3	276	Jun 19 2002	Oct 6, 2003	12.9° – 13.3°	10.2° – 10.4°
Bamboung MPA	Senegal	30	432	Mar 11, 2003	Oct 16, 2012	13.75° – 13.84°	16.5° – 16.56°
Banc d’Arguin National Park	Mauritania	3	86	May 7, 2008	May 28, 2010	19.4° – 20.8°	16° – 16.8°
Urok Island MPA	Guinea-Bissau	3	378	Nov 1, 2011	May 24, 2013	11.4° – 11.6°	15.9° – 16.1°

**Table 2.** List of the fish taxa collected in the PPEAO experimental fishing dataset, ordered by family, with their abundance per country (total number of individuals).

**Family**	**Taxon**	**Côte d'Ivoire**	**Guinea**	**Guinea Bissau**	**Gambia**	**Senegal**	**Mauritania**	**Mali**
Acanthuridae	*Acanthurus monroviae* Steindachner, 1876	24		19		11		
Albulidae	*Albula vulpes* (Linnaeus, 1758)	2		58		14		
Alestidae	*Alestes baremoze* (Joannis, 1835)				15			8
Alestidae	*Alestes dentex* (Linnaeus, 1758)				2			
Alestidae	*Brycinus leuciscus* (Günther, 1867)							451
Alestidae	*Brycinus macrolepidotus* Valenciennes, 1850	27	23					36
Alestidae	*Brycinus nurse* (Rüppell, 1832)	3			8			251
Alestidae	*Bryconalestes longipinnis* (Günther, 1864)	47	6					
Alestidae	*Hydrocynus brevis* Günther, 1864				1			1
Alestidae	*Hydrocynus forskahlii* (Cuvier, 1819)		27					992
Antennariidae	*Antennarius pardalis* (Valenciennes, 1837)			6		26		
Antennariidae	*Antennarius striatus* (Shaw, 1794)	139						
Apogonidae	*Apogon imberbis* (Linnaeus, 1758)	31						
Ariidae	*Carlarius heudelotii* (Valenciennes, 1840)		157		58	12	73	
Ariidae	*Carlarius latiscutatus* (Günther, 1864)	17	69	223	498	2,305	2	
Ariidae	*Carlarius parkii* (Günther, 1864)		15	83	121	946	246	
Atherinidae	*Atherina* sp.					863		
Bagridae	*Auchenoglanis occidentalis* (Valenciennes, 1840)							1
Bagridae	*Bagrus bajad* (Forsskål, 1775)							3
Bagridae	*Bagrus docmak* (Forsskål, 1775)							1
Batrachoididae	*Batrachoides liberiensis* (Steindachner, 1867)		4	1	12	293		
Batrachoididae	*Halobatrachus didactylus* (Bloch & Schneider, 1801)						7	
Belonidae	*Ablennes hians* (Valenciennes, 1846)			9		2	2	
Belonidae	*Belonidae* gen. sp.						5	
Belonidae	*Strongylura senegalensis* (Valenciennes, 1846)	67	2		129	257	5	
Belonidae	*Tylosurus acus* (Lacepède, 1803)			2	1	5	7	
Belonidae	*Tylosurus crocodilus* (Péron & Lesueur, 1821)		1			47		
Blenniidae	*Blennius* sp.					3		
Blenniidae	*Hypleurochilus langi* (Fowler, 1923)					4		
Blenniidae	*Parablennius goreensis* (Valenciennes, 1836)	1						
Bothidae	*Arnoglossus imperialis* (Rafinesque, 1810)			1				
Carangidae	*Alectis alexandrina* (Geoffroy Saint-Hilaire, 1817)	10				38	13	
Carangidae	*Campogramma glaycos* (Lacepède, 1801)						14	
Carangidae	*Caranx* sp.					2		
Carangidae	*Caranx crysos* (Mitchill, 1815)					1		
Carangidae	*Caranx hippos* (Linnaeus, 1766)	1,112	71	24	19	87		
Carangidae	*Caranx rhonchus* Geoffroy Saint-Hilaire, 1817	26				38	73	
Carangidae	*Caranx senegallus* Cuvier, 1833	2,617	220	8	79	92		
Carangidae	*Chloroscombrus chrysurus* (Linnaeus, 1766)	14,147	3,793	2,729	170	16,098	3,419	
Carangidae	*Hemicaranx bicolor* (Günther, 1860)	1	11		41	15		
Carangidae	*Lichia amia* (Linnaeus, 1758)	4	2	2	1	27	1	
Carangidae	*Selene dorsalis* (Gill, 1863)	1,698	4	1		15	2	
Carangidae	*Trachinotus ovatus* (Linnaeus, 1758)	97				2		
Carangidae	*Trachinotus teraia* Cuvier, 1832	399	40	1	23	143		
Carangidae	*Trachurus trecae* Cadenat, 1950					87	34	
Carcharhinidae	*Carcharhinus leucas* (Valenciennes, 1839)					2		
Carcharhinidae	*Carcharhinus limbatus* (Valenciennes, 1839)			2				
Carcharhinidae	*Rhizoprionodon acutus* (Rüppell, 1837)		2	38			6	
Chaetodontidae	*Chaetodon hoefleri* Steindachner, 1881					4	1	
Cichlidae	*Chromidotilapia guentheri* (Sauvage, 1882)	1				1		4
Cichlidae	*Coptodon zillii* (Gervais, 1848)							39
Cichlidae	*Coptodon guineensis* (Günther, 1862)	2,013	10	5	117	1,127		
Cichlidae	*Hemichromis bimaculatus* Gill, 1862							101
Cichlidae	*Hemichromis fasciatus* Peters, 1857	525	15	1	262	1,357		130
Cichlidae	*Pelmatolapia mariae* (Boulenger, 1899)	51						
Cichlidae	*Sarotherodon galilaeus* (Linnaeus, 1758)				3			86
Cichlidae	*Sarotherodon melanotheron* Rüppell, 1852	1,692			474	35,343		
Cichlidae	*Tilapia brevimanus* Boulenger, 1911		2					
Cichlidae	*Tylochromis intermedius* (Boulenger, 1916)		44					
Cichlidae	*Tylochromis jentinki* (Steindachner, 1894)	1,142			4			
Cichlidae	*Tylochromis leonensis* Stiassny, 1989		2					
Citharinidae	*Citharinus citharus* (Geoffroy Saint-Hilaire, 1809)				1			1
Clariidae	*Clarias anguillaris* (Linnaeus, 1758)				2			3
Clariidae	*Heterobranchus bidorsalis* Geoffroy Saint-Hilaire, 1809				3			
Clariidae	*Heterobranchus isopterus* Bleeker, 1863	2						
Claroteidae	*Chrysichthys* sp.							5
Claroteidae	*Chrysichthys auratus* (Geoffroy Saint-Hilaire, 1809)	2,992						265
Claroteidae	*Chrysichthys johnelsi* Daget, 1959		90		52			
Claroteidae	*Chrysichthys maurus* (Valenciennes, 1840)	12,004	27		815			
Claroteidae	*Chrysichthys nigrodigitatus* (Lacepède, 1803)	8,197	21		151			36
Clupeidae	*Ethmalosa fimbriata* (Bowdich, 1825)	350,416	3,164	95	13,179	147,618	838	
Clupeidae	*Pellonula leonensis* Boulenger, 1916	1,774	1,962		889	2,909		16
Clupeidae	*Sardina pilchardus* (Walbaum, 1792)						2	
Clupeidae	*Sardinella aurita* Valenciennes, 1847	57				154	2,220	
Clupeidae	*Sardinella maderensis* (Lowe, 1838)	35,490	3,907	417	3,829	179,803	4,867	
Congridae	*Uroconger lepturus* (Richardson, 1845)	2						
Cynoglossidae	*Cynoglossus* sp.						3	
Cynoglossidae	*Cynoglossus monodi* Chabanaud, 1949		5			3	1	
Cynoglossidae	*Cynoglossus senegalensis* (Kaup, 1858)	751	309	12	230	278		
Cyprinidae	*Enteromius macrops* (Boulenger, 1911)							293
Cyprinidae	*Labeo coubie* Rüppell, 1832							92
Cyprinidae	*Labeo parvus* Boulenger, 1902							7
Cyprinidae	*Labeo senegalensis* Valenciennes, 1842							71
Dactylopteridae	*Dactylopterus volitans* (Linnaeus, 1758)	1						
Danionidae	*Raiamas senegalensis* (Steindachner, 1870)							41
Dasyatidae	*Fontitrygon margarita* (Günther, 1870)		17	16	11	116		
Dasyatidae	*Fontitrygon margaritella* (Compagno & Roberts, 1984)	18		45	5	172		
Dasyatidae	*Fontitrygon ukpam* (Smith, 1863)				2			
Diodontidae	*Chilomycterus reticulatus* (Linnaeus, 1758)						1	
Diodontidae	*Chilomycterus spinosus* (Linnaeus, 1758)						7	
Diodontidae	*Diodon holocanthus* Linnaeus, 1758	1						
Distichodontidae	*Distichodus brevipinnis* Günther, 1864							45
Distichodontidae	*Distichodus rostratus* Günther, 1864							19
Drepaneidae	*Drepane africana* Osório, 1892	9	333	57	30	150		
Echeneidae	*Echeneis naucrates* Linnaeus, 1758	1	10	7		7	4	
Echeneidae	*Remora remora* (Linnaeus, 1758)					2		
Eleotridae	*Bostrychus africanus* (Steindachner, 1879)				38	14		
Eleotridae	*Butis koilomatodon* (Bleeker, 1849)				2	5		
Eleotridae	*Dormitator lebretonis* (Steindachner, 1870)				33			
Eleotridae	*Eleotris* sp.	1						
Eleotridae	*Eleotris daganensis* Steindachner, 1870	2						
Eleotridae	*Eleotris senegalensis* Steindachner, 1870	32	2					
Eleotridae	*Eleotris vittata* Duméril, 1861	33						
Elopidae	*Elops lacerta* Valenciennes, 1847	7,772	132	23	176	926		
Elopidae	*Elops senegalensis* Regan, 1909					93		
Engraulidae	*Engraulis encrasicolus* (Linnaeus, 1758)					2		
Ephippidae	*Chaetodipterus lippei* Steindachner, 1895		4	46	7	94		
Ephippidae	*Ephippus goreensis* Cuvier, 1831			7		19		
Exocoetidae	*Fodiator acutus* (Valenciennes, 1847)	42	5	1		73	4	
Fistulariidae	*Fistularia petimba* Lacepède, 1803	1						
Fistulariidae	*Fistularia tabacaria* Linnaeus, 1758					1	1	
Gerreidae	*Eucinostomus melanopterus* (Bleeker, 1863)	4,270	324	955	544	22,142	8	
Gerreidae	*Gerres* sp.					20		
Gerreidae	*Gerres nigri* Günther, 1859	10,646	1	307	216	16,088		
Glaucostegidae	*Glaucostegus cemiculus* (Geoffroy Saint-Hilaire, 1817)			1		30	1	
Gobiidae	*Awaous lateristriga* (Duméril, 1861)	2				1		
Gobiidae	*Bathygobius soporator* (Valenciennes, 1837)	2						
Gobiidae	*Gobiidae* gen. sp.					482		
Gobiidae	*Gobioides sagitta* (Günther, 1862)	8	3					
Gobiidae	*Gobionellus occidentalis* (Boulenger, 1909)	311			4	10		
Gobiidae	*Nematogobius maindroni* (Sauvage, 1880)				1			
Gobiidae	*Periophthalmus barbarus* (Linnaeus, 1766)		6		2	32		
Gobiidae	*Porogobius schlegelii* (Günther, 1861)	543	4	2	255	116		
Gobiidae	*Yongeichthys thomasi* (Boulenger, 1916)		12			1		
Gymnuridae	*Gymnura altavela* (Linnaeus, 1758)					2	3	
Gymnuridae	*Gymnura micrura* (Bloch & Schneider, 1801)		5	1	3	8		
Haemulidae	*Brachydeuterus auritus* (Valenciennes, 1832)	3,542	1,876		18	13,344		
Haemulidae	*Parakuhlia macrophthalmus* (Osório, 1893)					3		
Haemulidae	*Plectorhinchus macrolepis* (Boulenger, 1899)	22	8	7	19	164		
Haemulidae	*Plectorhinchus mediterraneus* (Guichenot, 1850)						32	
Haemulidae	*Pomadasys* sp.		1					
Haemulidae	*Pomadasys incisus* (Bowdich, 1825)	176		10	2	775	243	
Haemulidae	*Pomadasys jubelini* (Cuvier, 1830)	7,620	268		44	1,759	3	
Haemulidae	*Pomadasys perotaei* (Cuvier, 1830)		30	112	287	3,375		
Haemulidae	*Pomadasys rogerii* (Cuvier, 1830)	1				7	27	
Hemiramphidae	*Hemiramphus balao* Lesueur, 1821	17						
Hemiramphidae	*Hemiramphus brasiliensis* (Linnaeus, 1758)			1		102	16	
Hemiramphidae	*Hyporhamphus picarti* (Valenciennes, 1847)	24	2		3	11	3	
Hepsetidae	*Hepsetus odoe* (Bloch, 1794)	5			6			
Heterenchelyidae	*Pythonichthys macrurus* (Regan, 1912)	1						
Labridae	*Bodianus scrofa* (Valenciennes, 1839)						7	
Labridae	*Symphodus bailloni* (Valenciennes, 1839)						6	
Latidae	*Lates niloticus* (Linnaeus, 1758)		11					112
Lethrinidae	*Lethrinus atlanticus* Valenciennes, 1830	2		106				
Lobotidae	*Lobotes surinamensis* (Bloch, 1790)		8			1		
Lutjanidae	*Lutjanus dentatus* (Duméril, 1861)	3	10			3		
Lutjanidae	*Lutjanus goreensis* (Valenciennes, 1830)	38	3	1		122		
Mochokidae	*Synodontis* sp.							3
Mochokidae	*Synodontis bastiani* Daget, 1948	1						
Mochokidae	*Synodontis batensoda* Rüppell, 1832				629			
Mochokidae	*Synodontis filamentosus* Boulenger, 1901							7
Mochokidae	*Synodontis gambiensis* (Günther, 1864)				2,001			
Mochokidae	*Synodontis membranaceus* (Geoffroy Saint-Hilaire, 1809)							7
Mochokidae	*Synodontis ocellifer* Boulenger, 1900							11
Mochokidae	*Synodontis schall* (Bloch & Schneider, 1801)	1						44
Mochokidae	*Synodontis sorex* Günther, 1864							29
Monacanthidae	*Stephanolepis hispidus* (Linnaeus, 1766)			2		15	457	
Monodactylidae	*Monodactylus sebae* (Cuvier, 1829)	1,201	140	2	1,111	3,222		
Mormyridae	*Campylomormyrus tamandua* (Günther, 1864)							1
Mormyridae	*Hippopotamyrus pictus* (Marcusen, 1864)							2
Mormyridae	*Hyperopisus bebe* (Lacepède, 1803)				6			7
Mormyridae	*Marcusenius furcidens* (Pellegrin, 1920)	1						
Mormyridae	*Marcusenius senegalensis* (Steindachner, 1870)							10
Mormyridae	*Marcusenius thomasi* (Boulenger, 1916)		2					
Mormyridae	*Marcusenius ussheri* (Günther, 1867)	28						
Mormyridae	*Mormyrops anguilloides* (Linnaeus, 1758)		1		3			4
Mormyridae	*Mormyrus macrophthalmus* Günther, 1866							11
Mormyridae	*Mormyrus rume* Valenciennes, 1847							12
Mormyridae	*Petrocephalus ansorgii* Boulenger, 1903							8
Mormyridae	*Petrocephalus bane* (Lacepède, 1803)				36	3		
Mormyridae	*Petrocephalus bovei* (Valenciennes, 1847)	13						67
Mormyridae	*Petrocephalus soudanensis* Bigorne & Paugy, 1990							76
Mormyridae	*Petrocephalus tenuicauda* (Steindachner, 1894)		2					
Moronidae	*Dicentrarchus punctatus* (Bloch, 1792)					37	16	
Mugilidae	*Chelon bandialensis* (Diouf, 1991)				1			
Mugilidae	*Chelon dumerili* (Steindachner, 1870)		8	100	6	10,455	1	
Mugilidae	*Liza* sp.				1	227		
Mugilidae	*Liza aurata* (Risso, 1810)						1	
Mugilidae	*Mugil* sp.					53		
Mugilidae	*Mugil bananensis* (Pellegrin, 1927)		3	21	6	1,160		
Mugilidae	*Mugil cephalus* Linnaeus, 1758		10		2	1,912		
Mugilidae	*Mugil curema* Valenciennes, 1836	156	17	61	3	2,143		
Mugilidae	*Neochelon falcipinnis* (Valenciennes, 1836)	136	894	23	384	3,206		
Mugilidae	*Parachelon grandisquamis* (Valenciennes, 1836)	715	879	11	2,086	2,367		
Mullidae	*Pseudupeneus prayensis* (Cuvier, 1829)	19				12	23	
Muraenidae	*Gymnothorax afer* Bloch, 1795	1						
Myliobatidae	*Aetomylaeus bovinus* (Geoffroy Saint-Hilaire, 1817)					2		
Myliobatidae	*Rhinoptera marginata* (Geoffroy Saint-Hilaire, 1817)					3	6	
Notopteridae	*Papyrocranus afer* (Günther, 1868)		9		2	5		
Ophichthidae	*Pisodonophis semicinctus* (Richardson, 1848)				3	42		
Paralichthyidae	*Citharichthys stampflii* (Steindachner, 1894)	10,659	112	17	56	150		
Paralichthyidae	*Syacium guineensis* (Bleeker, 1862)						1	
Platycephalidae	*Solitas gruveli* (Pellegrin, 1905)	1						
Polynemidae	*Galeoides decadactylus* (Bloch, 1795)	731	879	63	239	4,394	157	
Polynemidae	*Pentanemus quinquarius* (Linnaeus, 1758)	53	882		454	1		
Polynemidae	*Polydactylus quadrifilis* (Cuvier, 1829)	1,918	239		283	105		
Polypteridae	*Polypterus endlicherii* Heckel, 1847	5						
Polypteridae	*Polypterus senegalus* Cuvier, 1829				12			1
Pomacentridae	*Chromis chromis* (Linnaeus, 1758)	1						
Pomatomidae	*Pomatomus saltatrix* (Linnaeus, 1766)						1	
Priacanthidae	*Priacanthus arenatus* Cuvier, 1829	2						
Pristigasteridae	*Ilisha africana* (Bloch, 1795)	253	15,753	152	6,946	34,719	3	
Procatopodidae	*Aplocheilichthys spilauchen* (Duméril, 1861)				93	1,698		
Psettodidae	*Psettodes belcheri* Bennett, 1831			27		29	20	
Rajidae	*Raja undulata* Lacepède, 1802						1	
Rhachycentridae	*Rachycentron canadum* (Linnaeus, 1766)			1				
Rhinobatidae	*Rhinobatos rhinobatos* (Linnaeus, 1758)			1			2	
Rhinopteridae	*Rhinoptera bonasus* (Mitchill, 1815)			1		2		
Scaridae	*Nicholsina usta* (Valenciennes, 1840)					3		
Scaridae	*Scarus hoefleri* (Steindachner, 1881)	1		1		2		
Scaridae	*Sparisoma rubripinne* (Valenciennes, 1840)						1	
Schilbeidae	*Parailia pellucida* (Boulenger, 1901)	2,293						74
Schilbeidae	*Schilbe intermedius* Rüppell, 1832	72			327			16
Schilbeidae	*Schilbe mandibularis* (Günther, 1867)	616						
Schilbeidae	*Schilbe micropogon* (Trewavas, 1943)		1					
Schilbeidae	*Schilbe mystus* (Linnaeus, 1758)							552
Schilbeidae	*Siluranodon auritus* (Geoffroy Saint-Hilaire, 1809)							1
Sciaenidae	*Argyrosomus regius* (Asso y del Rio 1801)					33	9	
Sciaenidae	*Pseudotolithus elongatus* (Bowdich, 1825)	4,963	6,304	1	21,342	2,141		
Sciaenidae	*Pseudotolithus epipercus* (Bleeker, 1863)		35					
Sciaenidae	*Pseudotolithus moorii* (Günther, 1865)		40		2	4		
Sciaenidae	*Pseudotolithus senegalensis* (Valenciennes, 1833)	89	5		181	51	1	
Sciaenidae	*Pseudotolithus senegallus* (Cuvier, 1830)		171	22	191	432		
Sciaenidae	*Pseudotolithus typus* Bleeker, 1863	2	456	7	66	63		
Sciaenidae	*Pteroscion peli* (Bleeker, 1863)	323	16		83	93		
Sciaenidae	*Sciaena umbra* Linnaeus, 1758						3	
Sciaenidae	*Umbrina canariensis* Valenciennes, 1843			1			1	
Sciaenidae	*Umbrina ronchus* Valenciennes, 1843				1			
Scombridae	*Orcynopsis unicolor* (Geoffroy Saint-Hilaire, 1817)					9		
Scombridae	*Scomberomorus tritor* (Cuvier, 1832)	171	152	22		253	21	
Scorpaenidae	*Scorpaena angolensis* Norman, 1935	1						
Scorpaenidae	*Scorpaena maderensis* Valenciennes, 1833					1		
Scorpaenidae	*Scorpaena scrofa* Linnaeus, 1758					10		
Scorpaenidae	*Scorpaena stephanica* Cadenat, 1943					1		
Serranidae	*Cephalopholis nigri* (Günther, 1859)	6						
Serranidae	*Epinephelus aeneus* (Geoffroy Saint-Hilaire, 1817)	663		4	1	207	6	
Serranidae	*Epinephelus marginatus* (Lowe, 1834)					1		
Serranidae	*Mycteroperca rubra* (Bloch, 1793)						2	
Serranidae	*Serranus cabrilla* (Linnaeus, 1758)					1	1	
Serranidae	*Serranus scriba* (Linnaeus, 1758)						9	
Soleidae	*Dagetichthys cadenati* (Chabanaud, 1948)		4	4	2	25		
Soleidae	*Dagetichthys lusitanicus* (de Brito Capello, 1868)	18				17	4	
Soleidae	*Dicologlossa cuneata* (Moreau, 1881)						1	
Soleidae	*Pegusa triophthalma* (Bleeker, 1863)	14		1		23	3	
Soleidae	*Solea* sp.					2		
Soleidae	*Solea senegalensis* Kaup, 1858						1	
Sparidae	*Boops boops* (Linnaeus, 1758)	1						
Sparidae	*Dentex canariensis* Steindachner, 1881	2					8	
Sparidae	*Diplodus bellottii* (Steindachner, 1882)					93	4,788	
Sparidae	*Diplodus sargus* (Linnaeus, 1758)					2	109	
Sparidae	*Diplodus vulgaris* (Geoffroy Saint-Hilaire, 1817)					6		
Sparidae	*Lithognathus mormyrus* (Linnaeus, 1758)					3	7	
Sparidae	*Pagellus bellottii* Steindachner, 1882						55	
Sparidae	*Pagrus auriga* Valenciennes, 1843						2	
Sparidae	*Pagrus caeruleostictus* (Valenciennes, 1830)	102		11		1	103	
Sparidae	*Sparus aurata* Linnaeus, 1758						1	
Sparidae	*Spondyliosoma cantharus* (Linnaeus, 1758)						62	
Sphyraenidae	*Sphyraena afra* Peters, 1844	496	134	5	20	98		
Sphyraenidae	*Sphyraena guachancho* Cuvier, 1829			1	7	48		
Stromateidae	*Stromateus fiatola* Linnaeus, 1758	3				4	2	
Syngnathidae	*Enneacampus kaupi* (Bleeker, 1863)	3						
Syngnathidae	*Hippocampus algiricus* Kaup, 1856			1		12	2	
Syngnathidae	*Syngnathidae* gen. sp.					1		
Syngnathidae	*Syngnathus pelagicus* Linnaeus, 1758					1	1	
Synodontidae	*Saurida brasiliensis* Norman, 1935	9						
Synodontidae	*Trachinocephalus myops* (Forster, 1801)					1		
Tetraodontidae	*Ephippion guttifer* (Bennett, 1831)	7	7	28	54	185	16	
Tetraodontidae	*Lagocephalus laevigatus* (Linnaeus, 1766)	57	11	1		28	1	
Tetraodontidae	*Sphoeroides spengleri* (Bloch, 1785)	4			1	66	93	
Tetraodontidae	*Tetraodon* sp.		9					
Tetraodontidae	*Tetraodon lineatus* Linnaeus, 1758							1
Torpedinidae	*Torpedo* sp.					2		
Torpedinidae	*Torpedo marmorata* Risso, 1810			1		4		
Torpedinidae	*Torpedo torpedo* (Linnaeus, 1758)						1	
Triakidae	*Mustelus mustelus* (Linnaeus, 1758)						2	
Trichiuridae	*Trichiurus lepturus* Linnaeus, 1758	513	383		27	138	3	
Triglidae	*Lepidotrigla cadmani* Regan, 1915	3						
Zeidae	*Zeus faber* Linnaeus, 1758		2					

**Table 3.** Number of fish families and taxa identified per country.

**Country**	**Surveys**	**Families**	**Taxa**
Côte d’Ivoire	73	60	113
Guinea	20	40	86
Guinea-Bissau	5	45	71
Gambia	13	40	92
Senegal	93	59	141
Mauritania	3	41	84
Mali	6	14	50

**Table 4.** List of taxa other than fish, ordered by phylum, class, order, family and species, with their total abundance (number of individuals)

**Phylum**	**Class**	**Order**	**Family**	**Taxon**	**Nt**
Arthropoda	Malacostraca	Decapoda	Galatheidae	*Galathea* sp.	8
Arthropoda	Malacostraca	Decapoda	Inachoididae	*Stenorhynchus* sp.	7
Arthropoda	Malacostraca	Decapoda	Menippidae	*Menippe nodifrons* Stimpson, 1859	1
Arthropoda	Malacostraca	Decapoda	Ocypodidae	*Afruca tangeri* (Eydoux, 1835)	2
Arthropoda	Malacostraca	Decapoda	Palaemonidae	*Macrobrachium vollenhoveni* (Herklots, 1857)	269
Arthropoda	Malacostraca	Decapoda	Palaemonidae	*Nematopalaemon hastatus* (Aurivillius, 1898)	1
Arthropoda	Malacostraca	Decapoda	Penaeidae	*Holthuispenaeopsis atlantica* (Balss, 1914)	1
Arthropoda	Malacostraca	Decapoda	Penaeidae	*Penaeus kerathurus* (Forskål, 1775)	16
Arthropoda	Malacostraca	Decapoda	Penaeidae	*Penaeus monodon* Fabricius, 1798	2
Arthropoda	Malacostraca	Decapoda	Penaeidae	*Penaeus notialis* Pérez Farfante, 1967	17,644
Arthropoda	Malacostraca	Decapoda	Portunidae	*Callinectes* sp.	1,714
Arthropoda	Malacostraca	Decapoda	Portunidae	*Callinectes amnicola* (Rochebrune, 1883)	5,395
Arthropoda	Malacostraca	Decapoda	Portunidae	*Callinectes pallidus* (Rochebrune, 1883)	1,242
Arthropoda	Malacostraca	Decapoda	Portunidae	*Sanquerus validus* (Herklots, 1851)	1
Arthropoda	Malacostraca	Decapoda	Sesarmidae	*Guinearma huzardi* (Desmarest, 1825)	4
Arthropoda	Malacostraca	Decapoda	Xanthidae	*Panopeus africanus* Milne-Edwards, 1867	17
Arthropoda	Malacostraca	Decapoda	unidentified	Decapoda	767
Arthropoda	Malacostraca	Stomatopoda	Squillidae	*Squilla mantis* (Linnaeus, 1758)	72
Mollusca	Bivalvia	Arcida	Arcidae	*Senilia senilis* (Linnaeus, 1758)	153
Mollusca	Cephalopoda	Myopsida	Loliginidae	*Loliginidae* gen. sp.	512
Mollusca	Cephalopoda	Myopsida	Loliginidae	*Loligo vulgaris* Lamarck, 1798	5
Mollusca	Cephalopoda	Octopoda	Octopodidae	*Octopus vulgaris* Cuvier, 1797	11
Mollusca	Cephalopoda	Sepiida	Sepiidae	*Sepia* sp.	778
Mollusca	Cephalopoda	Sepiida	Sepiidae	*Sepia bertheloti* d'Orbigny [in Férussac & d'Orbigny], 1835	15
Mollusca	Cephalopoda	Sepiida	Sepiolidae	*Sepiola* sp.	1
Mollusca	Gastropoda	Aplysiida	Aplysiidae	*Aplysia* sp.	5
Mollusca	Gastropoda	Neogastropoda	Melongenidae	*Pugilina morio* (Linnaeus, 1758)	66
Mollusca	Gastropoda	Neogastropoda	Muricidae	*Bolinus cornutus* (Linnaeus, 1758)	90
Mollusca	Gastropoda	Neogastropoda	Muricidae	*Hexaplex duplex* (Röding, 1798)	416
Mollusca	Gastropoda	Neogastropoda	Muricidae	*Murex* sp.	67
Mollusca	Gastropoda	Neogastropoda	Volutidae	*Cymbium* sp.	14
Mollusca	Gastropoda	Neogastropoda	Volutidae	*Cymbium cymbium* (Linnaeus, 1758)	2
Mollusca	Gastropoda	Neogastropoda	Volutidae	*Cymbium glans* (Gmelin, 1791)	3
Mollusca	Gastropoda	Neogastropoda	Volutidae	*Cymbium pepo* (Lightfoot, 1786)	14
Mollusca	Gastropoda	Nudibranchia	unidentified	Nudibranchia	1
Cnidaria	Scyphozoa	unidentified	unidentified	Scyphozoa	2,937

**Table 5.** List of the 20 main fish taxa identified in the Ebrié lagoon, with their total abundance (number of individuals) and their proportion (%). These taxa represent 97.5% of the total number of fish caught.

**Order**	**Family**	**Species**	**Nt**	**Proportion**
Clupeiformes	Clupeidae	*Ethmalosa fimbriata* (Bowdich, 1825)	350,416	70.22
Clupeiformes	Clupeidae	*Sardinella maderensis* (Lowe, 1838)	35,490	7.11
Perciformes	Carangidae	*Chloroscombrus chrysurus* (Linnaeus, 1766)	14,147	2.83
Siluriformes	Claroteidae	*Chrysichthys maurus* (Valenciennes, 1840)	12,004	2.41
Pleuronectiformes	Paralichthyidae	*Citharichthys stampflii* (Steindachner, 1894)	10,659	2.14
Perciformes	Gerreidae	*Gerres nigri* Günther, 1859	10,646	2.13
Siluriformes	Claroteidae	*Chrysichthys nigrodigitatus* (Lacepède, 1803)	8,197	1.64
Elopiformes	Elopidae	*Elops lacerta* Valenciennes, 1847	7,772	1.56
Perciformes	Haemulidae	*Pomadasys jubelini* (Cuvier, 1830)	7,620	1.53
Perciformes	Sciaenidae	*Pseudotolithus elongatus* (Bowdich, 1825)	4,963	0.99
Perciformes	Gerreidae	*Eucinostomus melanopterus* (Bleeker, 1863)	4,270	0.86
Perciformes	Haemulidae	*Brachydeuterus auritus* (Valenciennes, 1832)	3,542	0.71
Siluriformes	Claroteidae	*Chrysichthys auratus* (Geoffroy Saint-Hilaire, 1809)	2,992	0.60
Perciformes	Carangidae	*Caranx senegallus* Cuvier, 1833	2,617	0.52
Siluriformes	Schilbeidae	*Parailia pellucida* (Boulenger, 1901)	2,293	0.46
Cichliformes	Cichlidae	*Coptodon guineensis* (Günther, 1862)	2,013	0.40
Perciformes	Polynemidae	*Polydactylus quadrifilis* (Cuvier, 1829)	1,918	0.38
Clupeiformes	Clupeidae	*Pellonula leonensis* Boulenger, 1916	1,774	0.36
Perciformes	Carangidae	*Selene dorsalis* (Gill, 1863)	1,698	0.34
Cichliformes	Cichlidae	*Sarotherodon melanotheron* Rüppell, 1852	1,692	0.34

**Table 6.** List of the 22 main fish taxa identified in the Sine Saloum delta, with their total abundance (number of individuals) and their proportion (%). These taxa represent 98% of the total number of fish caught.

**Order**	**Family**	**Species**	**Nt**	**Proportion**
Clupeiformes	Clupeidae	*Sardinella maderensis* (Lowe, 1838)	119,980	34.56
Clupeiformes	Clupeidae	*Ethmalosa fimbriata* (Bowdich, 1825)	74,947	21.59
Cichliformes	Cichlidae	*Sarotherodon melanotheron* Rüppell, 1852	34,928	10.06
Clupeiformes	Pristigasteridae	*Ilisha africana* (Bloch, 1795)	30,015	8.65
Perciformes	Gerreidae	*Eucinostomus melanopterus* (Bleeker, 1863)	17,852	5.14
Perciformes	Gerreidae	*Gerres nigri* Günther, 1859	11,530	3.32
Perciformes	Haemulidae	*Brachydeuterus auritus* (Valenciennes, 1832)	11,115	3.20
Perciformes	Carangidae	*Chloroscombrus chrysurus* (Linnaeus, 1766)	8,102	2.33
Mugiliformes	Mugilidae	*Chelon dumerili* (Steindachner, 1870)	5,436	1.57
Perciformes	Polynemidae	*Galeoides decadactylus* (Bloch, 1795)	3,596	1.04
Clupeiformes	Clupeidae	*Pellonula leonensis* Boulenger, 1916	2,909	0.84
Perciformes	Haemulidae	*Pomadasys perotaei* (Cuvier, 1830)	2,612	0.75
Perciformes	Monodactylidae	*Monodactylus sebae* (Cuvier, 1829)	2,568	0.74
Mugiliformes	Mugilidae	*Neochelon falcipinnis* (Valenciennes, 1836)	2,048	0.59
Perciformes	Sciaenidae	*Pseudotolithus elongatus* (Bowdich, 1825)	2,002	0.58
Mugiliformes	Mugilidae	*Parachelon grandisquamis* (Valenciennes, 1836)	1,941	0.56
Mugiliformes	Mugilidae	*Mugil cephalus* Linnaeus, 1758	1,896	0.55
Cyprinodontiformes	Procatopodidae	*Aplocheilichthys spilauchen* (Duméril, 1861)	1,698	0.49
Perciformes	Haemulidae	*Pomadasys jubelini* (Cuvier, 1830)	1,530	0.44
Cichliformes	Cichlidae	*Hemichromis fasciatus* Peters, 1857	1,340	0.39
Mugiliformes	Mugilidae	*Mugil curema* Valenciennes, 1836	1,295	0.37
Cichliformes	Cichlidae	*Coptodon guineensis* (Günther, 1862)	952	0.27

**Table 7.** List of the 20 main fish taxa identified in the Fatala Estuary and the Dangara inlet, with their total abundance (number of individuals) and their proportion (%). These taxa represent 96% of the total number of fish caught.

**Order**	**Family**	**Species**	**Nt**	**Proportion**
Clupeiformes	Pristigasteridae	*Ilisha africana* (Bloch, 1795)	15,753	35.29
Perciformes	Sciaenidae	*Pseudotolithus elongatus* (Bowdich, 1825)	6,304	14.12
Clupeiformes	Clupeidae	*Sardinella maderensis* (Lowe, 1838)	3,907	8.75
Perciformes	Carangidae	*Chloroscombrus chrysurus* (Linnaeus, 1766)	3,793	8.50
Clupeiformes	Clupeidae	*Ethmalosa fimbriata* (Bowdich, 1825)	3,164	7.09
Clupeiformes	Clupeidae	*Pellonula leonensis* Boulenger, 1916	1,962	4.40
Perciformes	Haemulidae	*Brachydeuterus auritus* (Valenciennes, 1832)	1,876	4.20
Mugiliformes	Mugilidae	*Neochelon falcipinnis* (Valenciennes, 1836)	894	2.00
Perciformes	Polynemidae	*Pentanemus quinquarius* (Linnaeus, 1758)	882	1.98
Perciformes	Polynemidae	*Galeoides decadactylus* (Bloch, 1795)	879	1.97
Mugiliformes	Mugilidae	*Parachelon grandisquamis* (Valenciennes, 1836)	879	1.97
Perciformes	Sciaenidae	*Pseudotolithus typus* Bleeker, 1863	456	1.02
Scombriformes	Trichiuridae	*Trichiurus lepturus* Linnaeus, 1758	383	0.86
Perciformes	Drepaneidae	*Drepane africana* Osório, 1892	333	0.75
Perciformes	Gerreidae	*Eucinostomus melanopterus* (Bleeker, 1863)	324	0.73
Pleuronectiformes	Cynoglossidae	*Cynoglossus senegalensis* (Kaup, 1858)	309	0.69
Perciformes	Haemulidae	*Pomadasys jubelini* (Cuvier, 1830)	268	0.60
Perciformes	Polynemidae	*Polydactylus quadrifilis* (Cuvier, 1829)	239	0.54
Perciformes	Carangidae	*Caranx senegallus* Cuvier, 1833	220	0.49
Perciformes	Sciaenidae	*Pseudotolithus senegallus* (Cuvier, 1830)	171	0.38

**Table 8.** List of the 20 main fish taxa identified in the Bijagos Archipelago and the Rio Grande de Buba during the 1993 survey, with their total abundance (number of individuals) and their proportion (%). These taxa represent 97.5% of the total number of fish caught.

**Order**	**Family**	**Species**	**Nt**	**Proportion**
Perciformes	Carangidae	*Chloroscombrus chrysurus* (Linnaeus, 1766)	2,727	51.50
Perciformes	Gerreidae	*Eucinostomus melanopterus* (Bleeker, 1863)	931	17.58
Clupeiformes	Clupeidae	*Sardinella maderensis* (Lowe, 1838)	407	7.69
Perciformes	Gerreidae	*Gerres nigri* Günther, 1859	286	5.40
Clupeiformes	Pristigasteridae	*Ilisha africana* (Bloch, 1795)	112	2.12
Perciformes	Lethrinidae	*Lethrinus atlanticus* Valenciennes, 1830	105	1.98
Mugiliformes	Mugilidae	*Chelon dumerili* (Steindachner, 1870)	96	1.81
Clupeiformes	Clupeidae	*Ethmalosa fimbriata* (Bowdich, 1825)	74	1.40
Perciformes	Haemulidae	*Pomadasys perotaei* (Cuvier, 1830)	72	1.36
Albuliformes	Albulidae	*Albula vulpes* (Linnaeus, 1758)	50	0.94
Perciformes	Drepaneidae	*Drepane africana* Osório, 1892	49	0.93
Perciformes	Polynemidae	*Galeoides decadactylus* (Bloch, 1795)	47	0.89
Myliobatiformes	Dasyatidae	*Fontitrygon margaritella* (Compagno & Roberts, 1984)	45	0.85
Mugiliformes	Mugilidae	*Mugil curema* Valenciennes, 1836	42	0.79
Tetraodontiformes	Tetraodontidae	*Ephippion guttifer* (Bennett, 1831)	28	0.53
Scombriformes	Scombridae	*Scomberomorus tritor* (Cuvier, 1832)	20	0.38
Perciformes	Acanthuridae	*Acanthurus monroviae* Steindachner, 1876	19	0.36
Elopiformes	Elopidae	*Elops lacerta* Valenciennes, 1847	19	0.36
Perciformes	Ephippidae	*Chaetodipterus lippei* Steindachner, 1895	17	0.32
Pleuronectiformes	Paralichthyidae	*Citharichthys stampflii* (Steindachner, 1894)	16	0.30

**Table 9.** List of the 20 main fish taxa identified in the Gambia estuary, with their total abundance (number of individuals) and their proportion (%). These taxa represent 95% of the total number of fish caught.

**Order**	**Family**	**Species**	**Nt**	**Proportion**
Perciformes	Sciaenidae	*Pseudotolithus elongatus* (Bowdich, 1825)	21,342	35.83
Clupeiformes	Clupeidae	*Ethmalosa fimbriata* (Bowdich, 1825)	13,179	22.13
Clupeiformes	Pristigasteridae	*Ilisha africana* (Bloch, 1795)	6,946	11.66
Clupeiformes	Clupeidae	*Sardinella maderensis* (Lowe, 1838)	3,829	6.43
Mugiliformes	Mugilidae	*Parachelon grandisquamis* (Valenciennes, 1836)	2,086	3.50
Siluriformes	Mochokidae	*Synodontis gambiensis* (Günther, 1864)	2,001	3.36
Perciformes	Monodactylidae	*Monodactylus sebae* (Cuvier, 1829)	1,111	1.87
Clupeiformes	Clupeidae	*Pellonula leonensis* Boulenger, 1916	889	1.49
Siluriformes	Claroteidae	*Chrysichthys maurus* (Valenciennes, 1840)	815	1.37
Siluriformes	Mochokidae	*Synodontis batensoda* Rüppell, 1832	629	1.06
Perciformes	Gerreidae	*Eucinostomus melanopterus* (Bleeker, 1863)	544	0.91
Siluriformes	Ariidae	*Carlarius latiscutatus* (Günther, 1864)	498	0.84
Cichliformes	Cichlidae	*Sarotherodon melanotheron* Rüppell, 1852	474	0.80
Perciformes	Polynemidae	*Pentanemus quinquarius* (Linnaeus, 1758)	454	0.76
Mugiliformes	Mugilidae	*Neochelon falcipinnis* (Valenciennes, 1836)	384	0.64
Siluriformes	Schilbeidae	*Schilbe intermedius* Rüppell, 1832	327	0.55
Perciformes	Haemulidae	*Pomadasys perotaei* (Cuvier, 1830)	287	0.48
Perciformes	Polynemidae	*Polydactylus quadrifilis* (Cuvier, 1829)	283	0.48
Cichliformes	Cichlidae	*Hemichromis fasciatus* Peters, 1857	262	0.44
Gobiiformes	Gobiidae	*Porogobius schlegelii* (Günther, 1861)	255	0.43

**Table 10.** List of the 20 main fish taxa identified in Manantali and Sélingué reservoirs, with their total abundance (number of individuals) and their proportion (%). These taxa represented 94.2% of the total number of fish caught.

**Order**	**Family**	**Species**	**Nt**	**Proportion**
Characiformes	Alestidae	*Hydrocynus forskahlii* (Cuvier, 1819)	992	24.47
Siluriformes	Schilbeidae	*Schilbe mystus* (Linnaeus, 1758)	552	13.62
Characiformes	Alestidae	*Brycinus leuciscus* (Günther, 1867)	451	11.12
Cypriniformes	Cyprinidae	*Enteromius macrops* (Boulenger, 1911)	293	7.23
Siluriformes	Claroteidae	*Chrysichthys auratus* (Geoffroy Saint-Hilaire, 1809)	265	6.54
Characiformes	Alestidae	*Brycinus nurse* (Rüppell, 1832)	251	6.19
Cichliformes	Cichlidae	*Hemichromis fasciatus* Peters, 1857	130	3.21
Perciformes	Latidae	*Lates niloticus* (Linnaeus, 1758)	112	2.76
Cichliformes	Cichlidae	*Hemichromis bimaculatus* Gill, 1862	101	2.49
Cypriniformes	Cyprinidae	*Labeo coubie* Rüppell, 1832	92	2.27
Cichliformes	Cichlidae	*Sarotherodon galilaeus* (Linnaeus, 1758)	86	2.12
Osteoglossiformes	Mormyridae	*Petrocephalus soudanensis* Bigorne & Paugy, 1990	76	1.87
Siluriformes	Schilbeidae	*Parailia pellucida* (Boulenger, 1901)	74	1.83
Cypriniformes	Cyprinidae	*Labeo senegalensis* Valenciennes, 1842	71	1.75
Osteoglossiformes	Mormyridae	*Petrocephalus bovei* (Valenciennes, 1847)	67	1.65
Characiformes	Distichodontidae	*Distichodus brevipinnis* Günther, 1864	45	1.11
Siluriformes	Mochokidae	*Synodontis schall* (Bloch & Schneider, 1801)	44	1.09
Cypriniformes	Danionidae	*Raiamas senegalensis* (Steindachner, 1870)	41	1.01
Cichliformes	Cichlidae	Coptodon zillii (Gervais, 1848)	39	0.96
Siluriformes	Claroteidae	*Chrysichthys nigrodigitatus* (Lacepède, 1803)	36	0.89

**Table 11.** List of the 20 main fish taxa identified in the Bamboung MPA and its surroundings, with their total abundance (number of individuals) and their proportion (%). These taxa represent 98.6% of the total number of fish caught.

**Order**	**Family**	**Species**	**Nt**	**Proportion**
Clupeiformes	Clupeidae	*Ethmalosa fimbriata* (Bowdich, 1825)	72,671	41.97
Clupeiformes	Clupeidae	*Sardinella maderensis* (Lowe, 1838)	59,823	34.55
Perciformes	Carangidae	*Chloroscombrus chrysurus* (Linnaeus, 1766)	7,996	4.62
Mugiliformes	Mugilidae	*Chelon dumerili* (Steindachner, 1870)	5,019	2.90
Clupeiformes	Pristigasteridae	*Ilisha africana* (Bloch, 1795)	4,704	2.72
Perciformes	Gerreidae	*Gerres nigri* Günther, 1859	4,558	2.63
Perciformes	Gerreidae	*Eucinostomus melanopterus* (Bleeker, 1863)	4,290	2.48
Perciformes	Haemulidae	*Brachydeuterus auritus* (Valenciennes, 1832)	2,229	1.29
Siluriformes	Ariidae	*Carlarius latiscutatus* (Günther, 1864)	2,124	1.23
Mugiliformes	Mugilidae	*Neochelon falcipinnis* (Valenciennes, 1836)	1,158	0.67
Mugiliformes	Mugilidae	*Mugil curema* Valenciennes, 1836	848	0.49
Siluriformes	Ariidae	*Carlarius parkii* (Günther, 1864)	842	0.49
Perciformes	Polynemidae	*Galeoides decadactylus* (Bloch, 1795)	798	0.46
Perciformes	Haemulidae	*Pomadasys perotaei* (Cuvier, 1830)	763	0.44
Perciformes	Monodactylidae	*Monodactylus sebae* (Cuvier, 1829)	654	0.38
Perciformes	Haemulidae	*Pomadasys incisus* (Bowdich, 1825)	540	0.31
Elopiformes	Elopidae	*Elops lacerta* Valenciennes, 1847	478	0.28
Mugiliformes	Mugilidae	*Parachelon grandisquamis* (Valenciennes, 1836)	426	0.25
Cichliformes	Cichlidae	*Sarotherodon melanotheron* Rüppell, 1852	415	0.24
Mugiliformes	Mugilidae	*Mugil bananensis* (Pellegrin, 1927)	402	0.23

**Table 12.** List of the 20 main fish taxa identified in the Banc d’Arguin National Park, with their total abundance (number of individuals) and their proportion (%). These taxa represent 98.4% of the total number of fish caught.

**Order**	**Family**	**Species**	**Nt**	**Proportion**
Clupeiformes	Clupeidae	*Sardinella maderensis* (Lowe, 1838)	4,867	26.72
Perciformes	Sparidae	*Diplodus bellottii* (Steindachner, 1882)	4,788	26.29
Perciformes	Carangidae	*Chloroscombrus chrysurus* (Linnaeus, 1766)	3,419	18.77
Clupeiformes	Clupeidae	*Sardinella aurita* Valenciennes, 1847	2,220	12.19
Clupeiformes	Clupeidae	*Ethmalosa fimbriata* (Bowdich, 1825)	838	4.60
Tetraodontiformes	Monacanthidae	*Stephanolepis hispidus* (Linnaeus, 1766)	457	2.51
Siluriformes	Ariidae	*Carlarius parkii* (Günther, 1864)	246	1.35
Perciformes	Haemulidae	*Pomadasys incisus* (Bowdich, 1825)	243	1.33
Perciformes	Polynemidae	*Galeoides decadactylus* (Bloch, 1795)	157	0.86
Perciformes	Sparidae	*Diplodus sargus* (Linnaeus, 1758)	109	0.60
Perciformes	Sparidae	*Pagrus caeruleostictus* (Valenciennes, 1830)	103	0.57
Tetraodontiformes	Tetraodontidae	*Sphoeroides spengleri* (Bloch, 1785)	93	0.51
Siluriformes	Ariidae	*Carlarius heudelotii* (Valenciennes, 1840)	73	0.40
Perciformes	Carangidae	*Caranx rhonchus* Geoffroy Saint-Hilaire, 1817	73	0.40
Perciformes	Sparidae	*Spondyliosoma cantharus* (Linnaeus, 1758)	62	0.34
Perciformes	Sparidae	*Pagellus bellottii* Steindachner, 1882	55	0.30
Perciformes	Carangidae	*Trachurus trecae* Cadenat, 1950	34	0.19
Perciformes	Haemulidae	*Plectorhinchus mediterraneus* (Guichenot, 1850)	32	0.18
Perciformes	Haemulidae	*Pomadasys rogerii* (Cuvier, 1830)	27	0.15
Perciformes	Mullidae	*Pseudupeneus prayensis* (Cuvier, 1829)	23	0.13

**Table 13.** List of the 20 main fish taxa identified in the Urok Island MPA, with their total abundance (number of individuals) and their proportion (%). These taxa represent 89.5% of the total number of fish caught.

**Order**	**Family**	**Species**	**Nt**	**Proportion**
Siluriformes	Ariidae	*Carlarius latiscutatus* (Günther, 1864)	221	29.86
Siluriformes	Ariidae	*Carlarius parkii* (Günther, 1864)	73	9.86
Clupeiformes	Pristigasteridae	*Ilisha africana* (Bloch, 1795)	40	5.41
Perciformes	Haemulidae	*Pomadasys perotaei* (Cuvier, 1830)	40	5.41
Carcharhiniformes	Carcharhinidae	*Rhizoprionodon acutus* (Rüppell, 1837)	34	4.59
Perciformes	Ephippidae	*Chaetodipterus lippei* Steindachner, 1895	29	3.92
Perciformes	Gerreidae	*Eucinostomus melanopterus* (Bleeker, 1863)	24	3.24
Perciformes	Carangidae	*Caranx hippos* (Linnaeus, 1766)	22	2.97
Pleuronectiformes	Psettodidae	*Psettodes belcheri* Bennett, 1831	22	2.97
Clupeiformes	Clupeidae	*Ethmalosa fimbriata* (Bowdich, 1825)	21	2.84
Perciformes	Gerreidae	*Gerres nigri* Günther, 1859	21	2.84
Perciformes	Sciaenidae	*Pseudotolithus senegallus* (Cuvier, 1830)	20	2.70
Mugiliformes	Mugilidae	*Mugil curema* Valenciennes, 1836	19	2.57
Perciformes	Polynemidae	*Galeoides decadactylus* (Bloch, 1795)	16	2.16
Mugiliformes	Mugilidae	*Neochelon falcipinnis* (Valenciennes, 1836)	14	1.89
Mugiliformes	Mugilidae	*Mugil bananensis* (Pellegrin, 1927)	11	1.49
Clupeiformes	Clupeidae	*Sardinella maderensis* (Lowe, 1838)	10	1.35
Pleuronectiformes	Cynoglossidae	*Cynoglossus senegalensis* (Kaup, 1858)	9	1.22
Albuliformes	Albulidae	*Albula vulpes* (Linnaeus, 1758)	8	1.08
Perciformes	Drepaneidae	*Drepane africana* Osório, 1892	8	1.08
